# Multiblock Metabolomics Responses of the Diatom *Phaeodactylum tricornutum* Under Benthic and Planktonic Culture Conditions

**DOI:** 10.3390/md23080314

**Published:** 2025-07-31

**Authors:** Andrea Castaldi, Mohamed Nawfal Triba, Laurence Le Moyec, Cédric Hubas, Gaël Le Pennec, Marie-Lise Bourguet-Kondracki

**Affiliations:** 1 Molécules de Communication et Adaptation des Microorganismes, UMR 7245 CNRS, Muséum National d’Histoire Naturelle, 57 rue Cuvier (CP54), 75005 Paris, France; andrea.castaldi@inrae.fr (A.C.); laurence.lemoyec@univ-evry.fr (L.L.M.); 2 Laboratoire de Biotechnologie et Chimie Marines, Université Bretagne Sud, EMR CNRS 6076, IUEM, 56100 Lorient, France; 3 CB3S (Chimie Bioorganique, Biophysique, Biomatériaux pour la Santé)—UMR 7244 CNRS, Université Paris 13, 74 rue Marcel Cachin, 93017 Bobigny Cedex, France; mohamed.triba@univ-paris13.fr; 4 Université d’Evry Val d’Essonne-Paris Saclay, Boulevard F. Mitterrand, 91025 Evry, France; 5 Muséum National d’Histoire Naturelle, UMR BOREA, MNHN-CNRS-UCN-UPMC-IRD-UA, Station Marine de Concarneau, Concarneau, 75005 Paris, France; cedric.hubas@mnhn.fr

**Keywords:** *Phaeodactylum tricornutum*, metabolomics, LC-MS/MS, GC-MS, NMR, multivariate statistical analyses

## Abstract

This study investigates the metabolic responses of the model diatom *Phaeodactylum tricornutum* under different growth conditions, comparing benthic (adherent) and planktonic states. Using a multiblock metabolomics approach combining LC-HRMS^2^, NMR, and GC-MS techniques, we compared the metabolome of *P. tricornutum* cultivated on three laboratory substrates (glass, polystyrene, and polydimethylsiloxane) and under planktonic conditions. Our results revealed metabolic differences between adherent and planktonic cultures, particularly concerning the lipid and carbohydrate contents. Adherent cultures showed a metabolic profile with an increase in betaine lipids (DGTA/S), fatty acids (tetradecanoic and octadecenoic acids), and sugars (*myo*-inositol and ribose), suggesting modifications in membrane composition and lipid remodeling, which play a potential role in adhesion. In contrast, planktonic cultures displayed a higher content of cellobiose, specialized metabolites such as dihydroactinidiolide, quinic acid, catechol, and terpenes like phytol, confirming different membrane composition, energy storage capacity, osmoregulation, and stress adaptation. The adaptative strategies do not only concern adherent and planktonic states, but also different adherent culture conditions, with variations in lipid, amino acid, terpene, and carbohydrate contents depending on the physical properties of the support. Our results highlight the importance of metabolic adaptation in adhesion, which could explain the fouling process.

## 1. Introduction

Diatoms are unicellular photosynthetic organisms belonging to the phylum Heterokontophyta and are widely distributed in aquatic environments, including oceans, lakes, and rivers. As key members of phytoplankton, they account for about 20 to 25% of the Earth’s overall primary production, making them a vital component of oceanic ecosystems, as they serve as a source of energy and food for marine food webs, thus supporting the total productivity and biodiversity of the oceans [1]. These unicellular organisms produce a wide range of metabolites that possess bioactive properties with potential applications in pharmaceuticals, nutraceuticals, and industrial processes. Proteins, peptides, fatty acids, carotenoids, and polysaccharides isolated by diatoms have demonstrated various bioactivities ranging from ACE-inhibitory to antioxidant, anti-aging, anti-inflammatory, neuroprotective, antiproliferative, immunostimulant, antiviral, antihemolytic, and photoprotective effects [2]. Isolation and characterization of the compounds of interest have traditionally relied on targeted analytical approaches. However, their targeted identification can lead to the loss of metabolome-wide information and the degradation or instability of compounds prior to analyses [3]. With the rapid development of ‘omics’ approaches such as transcriptomics, proteomics, and metabolomics, new opportunities are emerging to provide a more comprehensive description of the diatom metabolome and its dynamic response to environmental conditions. In this context, integrated omics studies have already used *P. tricornutum* to highlight its ability to adapt its metabolism in response to stress conditions, particularly nitrogen and phosphorus deprivation, revealing rapid and flexible metabolic adaptation [4,5,6,7]. This well-established model diatom is used to study the physiology, evolution, and biochemistry of diatoms. Its ecological importance, phenotypic plasticity, and biotechnological potential make it especially valuable for studying the biofouling process [8,9]. The choice of this model diatom, despite its peculiar and different physiology from other diatoms, lies in its ability to grow easily under low light conditions and tolerate high pH levels. It is also the second marine diatom to have its genome sequenced [10,11]. In a maritime environment, the performance, durability, and safety of submerged surfaces largely depend on the effectiveness of the antifouling coatings applied to them. However, the long-term effectiveness of these coatings is limited, which allows undesirable fouling organisms to gradually colonize the surfaces [12,13,14]. Biofouling is a complex process that affects the physicochemical properties of surfaces, their hydrodynamics, and the chemical composition of extracellular polymeric substances [15,16,17]. Until now, few studies have really focused on the precise mechanisms by which these organisms adhere in the presence of these coatings, and the molecules involved in diatom adhesion are still not fully understood. Further research is needed to explore the potential of diatoms as biofouling agents and to further develop relevant strategies to prevent and control diatom biofouling [18,19]. Metabolic variations occurring during adhesion could help to elucidate the mechanisms of biofouling.

In this study, *P. tricornutum* was cultivated under controlled temperature and light conditions and in the presence of different substrates to better understand the molecular mechanisms of adhesion. Three easily obtainable laboratory substrates (glass = CG glass, polystyrene = CPT, polydimethylsiloxane = CPDMS) were selected to promote cell adhesion under static conditions, while a fourth planktonic condition (CP) was recreated to prevent substrate adhesion. These substrates differ significantly in physicochemical properties, including surface energy and wettability, ranging from hydrophilic (CG) to highly hydrophobic (CPT and CPDMS, respectively), which are known to influence cell–surface interactions; CPT is considered a rigid surface, and CPDMS a soft one [17]. The aim of this study was to explore how substrate-induced adhesion *versus* planktonic growth modulates the metabolic profile of *P. tricornutum*. To the best of our knowledge, this is the first study of the metabolic responses of the model diatom *P. tricornutum* grown in benthic compared to planktonic conditions.

## 2. Results

### 2.1. Multivariate Statistical Analyses of LC-HRMS^2^, GC-MS(CHCl_3_), GC-MS(MeOH), and ^1^H NMR Data

The PCA calculated with the LC-HRMS^2^ and the ^1^H NMR data already showed good separation between the supports, without any outliers. Regarding the PLS models, the predictive power of both the LC-MS and NMR matrices produced satisfactory statistical parameters Q^2^Y and AUROC, which were 0.76 and 0.99 for LC-HRMS^2^ and 0.73 and 0.99 for ^1^H NMR, respectively. Concerning GC-MS, PCA did not show complete separation between the supports, and the supervised PLS model did not reach correct statistical parameters.

**Figure 1:**
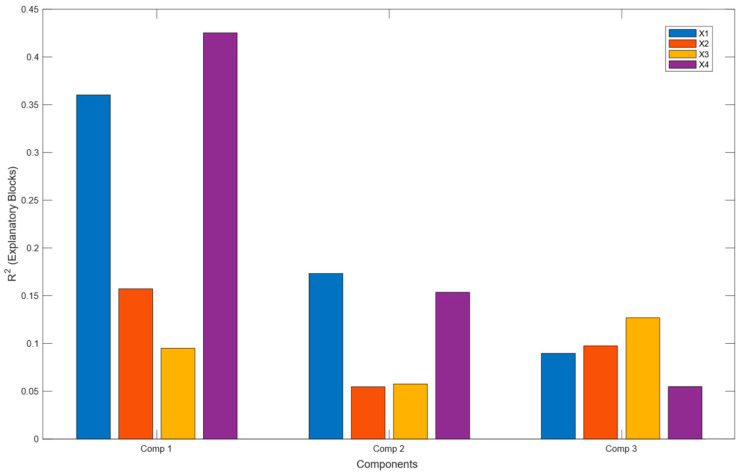
Explained variance (R^2^) per block for the three first components. Bar plot showing the proportion of variance explained (R^2^) in each data block by the first three components of the MB-PLS analysis. The different colors correspond to blocks X1, X2, X3, and X4.

A multiblock projection on latent structure (MBPLS) model was then constructed using the four matrices X1-X4. The R^2^Y value was 0.96 with calculation of the four components, the Q^2^Y value was evaluated at 0.76, and the AUROC was evaluated at 1. The contributions of each block to the three first axes of the model are presented in Figure 1. The lower contribution of the GC X2 and X3 matrices confirms the results obtained with the PLS model regarding these blocks, at least on the first axis. However, these matrices were kept in the model for their highly elucidating capacity.

**Figure 2:**
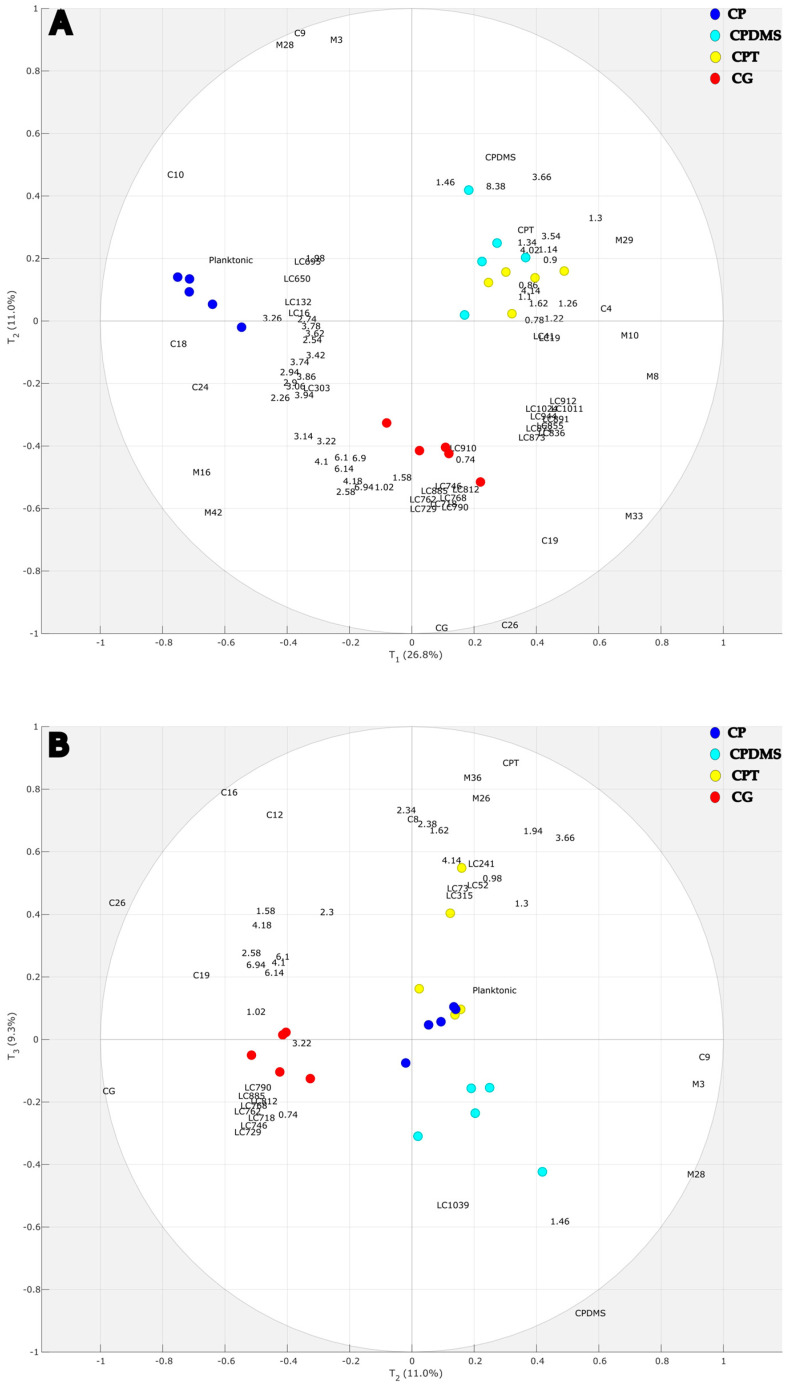
Biplots of the MBPLS model: score plots allow separations among groups of samples, reflecting both intra- and inter-group variability. Each dot corresponds to data from one sample colored according to the culture conditions (CG = glass, CPT = polystyrene, CPDMS = polydimethylsiloxane, CP = planktonic). Loading plots of most discriminant variables for each analytical technique computed in the MBPLS model with LC-HRMS^2^, GC-MS(MeOH), GC-MS(CHCl_3_), and ^1^H NMR data associated with the component score (T_1_–T_2_–T_3_). (**A**). The axes correspond to the scores of the first two components of the model (T_1_ and T_2_). (**B**). The axes correspond to the scores of the second and third components of the model (T_2_ and T_3_). The percentages represent the proportion of the total variance of the data explained by each component.

The biplot (Figure 2) highlights both group separation (score plot) and discriminant variables (loading plot). Figure 2A demonstrates good separation along the first predictive component (T_1_), which discriminates planktonic diatom samples (CP) from diatoms adhering to the three supports (CG, CPDMS, CPT). The second predictive component (T_2_) shows separation of the glass adherent culture (CG) from the other two adhering cultures (CPDMS and CPT) and the planktonic culture (CP). The biplot in Figure 2B shows the second and third predictive components (T_2_ and T_3_), demonstrating separation between CPT and CPDMS. The variables with the highest weights on the three components are presented in Figure 2 as labels in the plots, with the prefixes LC for X1, C for X2, and M for X3 and the chemical shift value for X4. Therefore, the variables closer to the sample group represent the increased metabolites in this group.

### 2.2. Discriminant Metabolites from X1 Matrix (LC-HRMS^2^) in the Multiblock Model

To display the total metabolome across culture conditions and highlight structural similarities, a comprehensive feature-based molecular network (FBMN) was generated. The resulting FBMN comprised 1049 nodes (*m*/*z* values) and 110 clusters of at least two nodes (Figure S1). The final ion identity molecular network (IIMN) comprised a total of 933 nodes (*m*/*z* values) and 90 clusters of at least two nodes. Each node was made up of four parts representing the average of the precursor ion intensity for the four cultures conditions. The size of the nodes was proportional to the average precursor ion intensity of the total samples. The width of the edges corresponded to the cosine similarity score between nodes. The shapes of the nodes corresponded to different categories: collapsed nodes from one or several adducts, ion identity nodes representing single adduct identifications, and feature nodes that did not have identified adducts from the MzMine pipeline. To improve the annotations of chemical families based on ClassyFire ontologies, ConCISE generated a single csv file containing the complete chemical hierarchy of consensus annotations derived from the GNPS spectral library and the CANOPUS annotations (Sirius v5.8.6). This integration allowed for the incorporation of information from NPClassifier and/or ClassyFire ontologies, with node border colors assigned based on their respective NP families (Figure S2) or ClassyFire ontologies (Figure S3). Multiple pipelines were utilized for compound annotation. The GNPS spectral library tentatively annotated 126 metabolites. Of the 933 *m*/*z* nodes, initially labelled in black, a total of 560 *m*/*z* (labeled in blue) were tentatively annotated within the Eukaryota domain using tima-R 2.10. More consistent annotation was achieved for 29 of these *m*/*z* values (labeled in red) within the Bacillariophyceae class, which belongs to the Bacillariophyta phylum (diatoms). For lipids, each discriminant node from the multivariate statistical analyses was subjected to final analysis of its original MS^2^ spectrum. Table 1 provides the retention time, accurate mass, and molecular formula, along with their respective Δ ppm error values. The MS/MS fragmentation patterns of interest are presented in the Supplementary Material (Figure S4).

Out of the LC-HRMS^2^ matrix, 16 *m*/*z* values were discriminant on T_1_ (Table 1). Among them, 11 *m*/*z* values were found to be increased in adherent cultures when compared to CP cultures, while five *m*/*z* values were increased in CP cultures when compared to adherent ones. These results are summarized in Figure 2A. The 11 *m*/*z* values increased in adherent cultures are one sugar (LC41), one dicarboxylic acid (LC19), and nine betaine lipids, in particular acylglycerolipids with an ether-linked quaternary amine alcohol moiety at the *sn*-3 position, constituting diacyl-glyceryl-hydroxymethyl-*N*,*N*,*N*-trimethyl-β-alanine (DGTA) or diacyl-glycerol-*N*,*N*,*N*-trimethylhomoserine (DGTS). All were characterized by the presence of diagnostic fragment ions at *m*/*z* 236.1498 (C_10_H_22_NO_5_^+^) and *m*/*z* 144.1025 (C_7_H_14_NO_2_^+^), and their fatty acids (FA) substituents were identified by the neutral loss of fatty acyl chains as acid (-RCOOH) and ketene (-R = C = O) derivatives [20]. These nine betaine lipids are LC836, LC855, LC872, LC873, LC891, LC912, LC944, LC1011, and LC1024. Of the five *m*/*z* values that were higher in CP cultures, only three molecules are tentatively annotated: a phenolic acid identified as catechol (LC16), dihydroactinidiolide, a monoterpenoid annotated from all pipelines (LC132), and quinic acid (LC303). A summary of the annotated features and chemical families is provided in the molecular network (Figure S5).

Out of the LC-HRMS^2^ matrix, eight *m*/*z* values were discriminant on the second predictive axis (T_2_). All of these eight *m*/*z* values (LC718, LC729, LC746, LC762, LC768, LC790, LC812, and LC885) were increased in CG cultures when compared to the three other cultures (Figure 2A and Table 1) and correspond to eight DGTS/As (Figure S6).

Finally, the third axis (T_3_) includes five discriminant *m*/*z* values. One of them (LC1039) was found to be higher in CPDMS adherent culture when compared to CPT (Figure 2B negative *y*-axis T_3_), while four *m*/*z* values (LC52, LC73, LC241, LC315) were found to be increased in CPT cell cultures when compared to CPDMS (Figure 2B, positive *y*-axis T_3_) (Table 1) (Figure S7).

### 2.3. Analyses of Matrices X2 GC-MS(CHCl_3_) and X3 GC-MS(MeOH) in the Multiblock Model

The discriminant variables of the GC-MS matrices on the predictive axis (T_1_) were ten *m*/*z* variables (Table 2 and Table 3). Of these, five *m*/*z* values were increased in adherent cultures when compared to CP cultures. These variables correspond to one SFA (C4), one MUFA (M33), and three carbohydrates (M8, M10, M29). The last five *m*/*z* values were increased in CP cultures when compared adherent cultures; two are carbohydrates (M16, M42), one is a PUFA (C10), one is a terpene (C18), and one is an oxylipin (C24).

Along the second axis, five *m*/*z* values were discriminant across the adherent cell cultures. Three *m*/*z* values were increased in CP, CPDMS, and CPT cultures when compared to CG cultures (Figure 2A). These *m*/*z* values were annotated as one branched alkane (C9), one polyol (M3), and one carbohydrate (M28). The two other *m*/*z* values were increased in CG adherent cultures when compared to the three others (Figure 2A), including two PUFAs (C19, C26).

The third axis allowed the separation of CPT cultures from CPDMS cultures with five *m*/*z* variables which were increased in CPT cultures compared to CPDMS cultures and were annotated as one branched alkane (C8), one MUFA (C12), one terpenoid (C16), and two carbohydrates (M26, M36).

### 2.4. Discriminant Metabolites from the X4 Matrix ^1^H-NMR in the Multiblock Model

The discriminant variables of the ^1^H NMR matrix on T_1_ were 21 resonances (Table 4). Among these, eleven regions were increased in adherent cultures when compared to CP cultures (Figure 2A). Analysis of the lipid classes presented particular challenges due to their proximity to polar groups, which are highly sensitive to the sample solvent. Assignments for these regions were attempted by comparison with literature data [21,22] and the results of the HSQC and TOCSY spectra (Figures S8–S11). These regions correspond to terminal methyl groups (δ 0.90 ppm), methylene chains (δ 1.30 ppm), and groups adjacent to ester bonds (δ 1.60 and 2.35 ppm), as well as signals associated with glycerophospholipids and/or glycerolipids (δ 3.54, 3.82, 4.01, and 4.10 ppm) and formate (δ 8.40 ppm). The observed HSQC values only partially matched the literature data. While the pattern of signals at δ 4.01 and 4.10 ppm indicated the characteristic glycerol backbone of lipids, the typical sn-2 position signal around 5 ppm was absent. The absence of a signal around 5 ppm was found only in lysophosphocholine (LPC) and sn-1,2 diacylglycerol (DAG). However, this does not explain the unusually low signal at δ 3.54 ppm, which is part of the same spin system and atypical for LPC or other glycerolipids. The region is characterized by significant overlapping, leading to low annotation confidence with putative attribution of functional groups. The last ten regions were increased in CP cultures when compared to adherent cultures: 2,3-dihydroxypropane-1-sulfonate (DHPS) and dimethylsulfoniopropionate (DMSP), carbohydrates, likely glucose or galactose, choline (δ 3.21 ppm), and a signal at δ 3.65 ppm that was attributed to free glycerol.

Along the second axis, 14 resonances were discriminant across the adherent cell cultures. Of these, lactate (δ 1.45 ppm) and glycerol (δ 3.65 ppm) were increased in CP, CPDMS, and CPT cultures compared to CG cultures (Figure 2A). The remaining 12 resonances were increased in CG adherent cultures when compared to the three others (Figure 2A): sterols (δ 0.74 ppm), amino acids such as valine (δ 1.01 ppm), isoleucine (δ 1.03 ppm), and proline (δ 2.32 ppm), choline (δ 3.21 ppm), fatty acid signals at δ 1.60 ppm (CH_2_ β-ester), δ 2.60 ppm (CH_2_ α-ester), and δ 4.12, 4.19 ppm (sulphoquinovosyldiacylglycerols SQDGs), and unassigned signals at δ 2.82, 6.10, and 6.92 ppm.

The third axis (T_3_) includes six resonances increased in CPT cultures when compared to CPDMS (Figure 2B): δ 0.90 ppm (terminal CH_3_), 1.30 ppm (CH_2_ chains), 1.60 ppm (CH_2_ β-ester), 2.35 ppm (CH_2_ α-ester), and δ 4.10 ppm (glycerophospholipids/glycerolipids).

## 3. Discussion

*Phaedactylum tricornutum* is a well-established model diatom with a described genome and chemistry that has been studied under various conditions, including nitrogen, phosphorus, and iron deprivation [4,23,24,25]. To date, transcriptomics and proteomics are the most commonly techniques used to analyze the biochemistry and physiology of this pelagic microalgae [26,27,28]. Some GC-MS studies [29,30] and LC-MS analyses [31,32] have also been reported. However, NMR metabolomics studies on diatoms are scarce [33,34], with no specific investigations of *P. tricornutum*. The aim of our study was to develop a metabolomics approach using three analytical techniques: LC-MS, ^1^H NMR, and GC-MS. This multiblock approach was developed in order to facilitate the analysis of metabolic variations under different culture conditions and to compare the metabolomes in planktonic *versus* adherent cultures.

The MBPLS model obtained classifies, with the first component, planktonic from adherent cultures, while adherent cultures are classified with the second and third components. The supervising factors of the MBPLS model were the four substrate compositions. However, the outcomes showed differentiation according to the physicochemical properties of these substrates. Therefore, regarding the properties of the supports, the second component classifies the metabolome of diatoms grown on a hydrophilic support (CG) from two hydrophobic supports (CPT and CPDMS). The third component classifies the two hydrophobic supports, one hard (CPT) and one soft (CPDMS).

Analysis of the metabolites involved in the separation of CP cultures of *P. tricornutum* from adherent cultures (CPT, CPDMS, and CG) suggests distinctive adaptive strategies related to membrane remodeling, energy storage, and osmoregulation.

The metabolomic differences identified between planktonic and adherent cultures highlight the modulation of lipid and carbohydrate constituents, suggesting particular strategies employed by *P. tricornutum* in its different culture conditions. In adherent cultures compared to planktonic cultures, the higher content of specific betaine lipids (DGTA/DGTS), including tetradecanoic acid (GC-MS), may have a key role in lipid remodeling [35], a crucial process for surface attachment, and consequently putative biofilm formation. It has been reported that the replacement of phosphocholine (PC) with betaine lipids such as DGTA and DGTS was considered a phosphorus-saving strategy [36,37]. This lipid remodeling was also observed in our LC-MS/MS and NMR results. The MUFA C18:1n-9, which increases in adherent cultures or decreases in the planktonic one in our study, was reported as a key intermediate in *P. tricornutum* lipid metabolism, particularly for biosynthesis of the PUFA eicosapentanoic acid (EPA) [38]. Therefore, a lower content in our planktonic cultures suggests that EPA metabolism might be modified when diatoms are maintained in pelagic conditions. In planktonic cultures, GC-MS analysis reveals increased PUFA C16:2n-6 and oxylipin C18:0;O3, which are involved in membrane fluidity and interspecies communication, respectively [39].

Several discriminant metabolites, abundant in the extracellular polymeric substances (EPS) of marine biofilms, were detected as discriminant variables. In adherent cultures, *myo*-inositol, known as a growth factor for heterotrophic bacteria, is increased and may support diatom adhesion [40,41]. In planktonic cultures, several carbohydrate metabolites were increased when compared to adherent diatoms. Among those, an unknown sugar as well as a disaccharide, glucose and/or galactose, was detected by NMR and identified as cellobiose by GC-MS [42]. Finally, ribose, which participates in RNA metabolism [43], was found to be higher in adherent cultures. These changes may reflect cell wall alterations or modified EPS secretion patterns during adhesion processes [44].

The increase of DMSP, choline, and DHPS in planktonic cultures supports the hypothesis of membrane adaptation to different environments. Indeed, DMSP and choline act as osmoprotectants [33,45], while DHPS serves as a carbon and sulfur reservoir [46]. Other discriminant metabolites increased in planktonic cultures when compared to adherent diatoms may participate in adaptation to culture conditions, including turbulent environments. Based on the hypothesis that turbulence produces different light exposure for planktonic diatoms than for adherent diatoms, this discrepancy may generate some metabolic changes. These metabolites include dihydroactinidiolide [47], a photosynthetic byproduct and signaling molecule, which was previously reported as being modulated in planktonic cells to adapt to light variations. Accordingly, phytol, a terpene linked to chlorophyll biosynthesis, enhances light harvesting and photoprotection, suggesting greater energy storage and chloroplast activity, as previously described [48,49]. The increase in catechol indicates a response to stressors like environmental fluctuations, aiding Fe(II)/Fe(III) acquisition and oxidative stress management [50]. Quinic acid, also increased in turbulent conditions, was previously reported as a salinity adaptation factor [51]. However, the higher content of formate in adherent cultures can be linked to a fermentative metabolism, which may occur due to lower light exposition [52].

The glass culture conditions can be separated from the three other conditions along the second axis. In addition, this classification can be also analyzed as the separation between a hydrophilic support (CG) and two hydrophobic supports (CPT and CPDMS). The discriminant metabolites, which are increased in CG when compared to CPT and CPDMS, are consistent with reprogramming of lipid metabolism and amino acid composition. DGTA/S, which were increased in CG cultures when compared to CPT and CPDMS cultures (T_2_ axis) presented shorter acyl chains than DGTA/S discriminating adherent cells from planktonic ones (T_1_ axis). Fatty acids detected by GC-MS and NMR are also increased when diatoms are grown on glass, suggesting modulation of lipid metabolism that could influence diatom membrane properties. Interestingly, glycerol and *N*-acetyl-D-glucosamine, known for their viscosity properties, are higher in cultures grown on a hydrophobic support, discriminating glass culture from the other ones. Conversely, SQDG and sterols are higher in CG culture compared to the other ones, imply a different mechanism of adhesion. Some amino acids were found to be increased in CG when compared to CPT and CPDMS cultures. Two of them (valine and isoleucine) participate in energy metabolism through the tricarboxylic acid (TCA) cycle, and proline is an osmoprotectant compound [53].

Interestingly, the cultures on the two hydrophobic supports can be discriminated using the third component of the MBPLS model, which corresponds to separation of hard (CPT) and soft (CPDMS) hydrophobic supports. The metabolic modifications also concern lipids, carbohydrate metabolism, and terpenoids, which are increased in CPT when compared to the other cultures. Palmitoleic acid, (9Z)-hexadec-9-enoic acid, is known to play a crucial role in the lipid composition and metabolism of *P. tricornutum* under different growth conditions [54]. The presence of the terpene isophytol-acetate, belonging to the phytyl fatty acid ester family, may reflect shifts in energy storage and membrane stabilization processes, as it has previously been associated with stress or senescence in plants [55]. Glucose and D-glucuronic acid were reported as being included in polysaccharidic molecules in diatoms [56]. The precise biological function of branched alkane 2-methyloctadecane remains unclear and requires further investigation. These metabolic modulations may reflect increased energy storage demand and differential mechanisms in cell adhesion in the CPT and CPDMS cultures.

Although no discriminant compound was shared across the multi-technique analyses of the different culture conditions, and the metabolomic profiles are not directly connected to the metabolic mechanisms involved in adhesion of *P. tricornutum*, their analysis (Tables S1–S3) may generate valuable insights for further investigation into adhesion-related processes, including membrane lipid composition and its resulting properties.

## 4. Materials and Methods

### 4.1. Microalgae Culture

*Phaeodactylum tricornutum* (AC590) was obtained from the Algobank Caen (University of Caen, Caen, France). The stock culture was cultivated in artificial seawater containing 2% f/2 medium and silica in 500 mL borosilicate glass Erlenmeyer flasks (Fisherbrand™, Waltham, MA, USA), at 20 °C, with a 12:12 h light:dark cycle. The stock culture was further synchronized for 40 h on a 24 h dark cycle, in the presence of antibiotics (penicillin G sodium salt, Sigma, streptomycin and chloramphenicol, Sigma, St. Louis, MO, USA). Cell seeding (10^6^ cells mL^−1^) was performed into soda-lime glass Petri dishes (Fisherbrand™) and polystyrene Petri dishes with and without a PDMS coating (diameter 15 cm) and for planktonic culture into 500 mL borosilicate glass Erlenmeyer flasks, maintained under bubbling conditions with 0.2 µm filtered, air and a 12:12 h light:dark cycle at 20 °C. All the cultures were individually collected after seven days, visual confluence was checked, and the cells were pelleted by centrifugation at 4 °C for 10 min at 6000 rpm in an Eppendorf centrifuge 5804 R. The resulting pellets were frozen in liquid nitrogen, then stored at −70 °C prior to lyophilization (HETO–Powerdry LL1500 freeze dryer, Thermo Scientific, Tehovec, Mukarov, Czech Republic) and extraction. The microalgae culture was repeated twice to obtain enough quantities for analyses, as follows: the first batch of 20 samples (five replicates for each culture condition: CG, CPT, CPDMS, and CP) was used for GC-MS analyses, and the second batch of an additional 20 samples was used for LC-ESI-HRMS^2^ and ^1^H NMR analyses. The extraction procedure with MeOH/CHCl_3_ (1:1), fractionation, and sample processing steps was performed identically to the established protocol described in [39]. Three successive elutions were performed, each with 6 mL of H_2_O, MeOH, and CHCl_3_. The MeOH (mid-polar) and CHCl_3_ (apolar) fractions were dried at room temperature with air filtered through 0.2 μm membrane filters. The polar fraction was discarded to minimize damage to the GC-MS and LC-MS syringe and column caused by excessive salt concentrations. The mid-polar and apolar fractions were subsequently analyzed by GC-MS. The mid-polar fraction was also analyzed by LC-MS and NMR spectroscopy. This extraction method was chosen to maximize metabolite detection by leveraging the complementary nature of the three analytical techniques.

### 4.2. Metabolomics

#### 4.2.1. Analytical Methods and Data Processing

##### Mass Spectrometry and Metabolite Annotations

LC-ESI-HRMS^2^ analyses were performed using an ultra-high performance liquid chromatography (UHPLC) system (Ultimate 3000 RSLC, Thermo Scientific, Waltham, MA, USA) coupled to a high-resolution electrospray ionization-quadrupole time-of-flight (ESI-Q-TOF) mass spectrometer (MaXis II ETD, Bruker Daltonics, Billerica, MA, USA). An Acclaim RSLC Polar Advantage II column (2.2 µm, 2.1 × 100 mm, Thermo Scientific, Waltham, MA, USA) was used for LC separation, with an injection volume of 4 μL and a flow rate of 0.3 mL.min^−1^. An isocratic flow of 1% B was maintained from 0 to 2 min (A: H_2_O + 0.10% formic acid, B: ACN + 0.08% formic acid), followed by a linear gradient from 1% to 100% B for 20 min and then 100% B over 2 min, then decreasing to 5% over 3 min, for a total run time of 25 min.

Mass spectrometry data acquisition was identical to that previously described, as well as detection, chromatogram construction, peak resolution, and the deisotoped module [57]. A duplicate peak filter module was applied (average filter mode, *m*/*z* tolerance: 10 ppm, retention time, RT tolerance: 0.3 min). Peak alignment was performed using the Ransac aligner algorithm (*m*/*z* tolerance at 10 ppm, absolute RT tolerance at 0.1 min). The aligned list was gap-filled using the peak finder algorithm (*m*/*z* tolerance at 10 ppm), with the RT tolerance set to 0.3 min and the intensity tolerance set to 0.5%. Blank subtraction was performed using quality control (QC) medium and QC blank solvent raw data, with a minimum of one detection in blanks, maximum ratio type, and quantification based on height. Finally, the peak list was filtered to exclude peaks without an associated MS/MS spectrum, outside the RT of 0.5–25.05 min, and within the 80 to 1300 *m*/*z* range. The metaCorrelate feature grouping was used with a minimum feature height and an intensity threshold for a correlation of 1.0 × 10^3^. An ion identity networking module was used to annotate the adducts and modifications with an *m*/*z* tolerance (intra-sample) of 0.0015 *m*/*z* or 10 ppm. The exported csv file was used to construct an X1 matrix, where each row represents a diatom sample, and each column represents a detected *m*/*z* value with relative feature intensity (1049 variables).

The ion-identity molecular networks were generated with identical parameters to the FBMN described in [57], with the addition of edge annotations. Public access to the collapsed ion molecular networks job for molecular networking is available at https://gnps.ucsd.edu/ProteoSAFe/status.jsp?task=3d688a78ced74bef860bb1ce6fbcf402 accessed on 24 May 2023. ConCISE was used with the same parameters as in [57]. To improve the reliability of annotations, a taxonomy-based reweighting phase [58] selected the top five choices (final rank) for each annotated node, considering the congruence of the biological sources described in the in silico Spectral Databases of Natural Products (ISDB) [59] and LOTUS database [60] at the level of species > genus > family. Sirius v5.8.6 [61] was employed to calculate feature raw formulas and to predict fragmentation patterns using the CSI: fingerID module. These predictions were cross-validated using the metabolomic pipeline from Metaboscape (v.5.0 (Bruker Daltonics, Bremen, Germany), including annotation from in-house and Natural Products Atlas (DB Version 2024_03 accessed on 24 March 2024) databases [62]. Cytoscape (ver. 3.10.0) software was used to analyze and display the molecular networking data [63].

##### GC–MS Analyses and Metabolite Annotations

The samples for GC-MS analyses were derivatized as follow: in the MeOH fractions, the functional groups (-OH, COOH and NH_2_) are converted into TMS derivatives. Fatty acids in the dried CHCl_3_ fractions undergo transesterification to yield the corresponding fatty acid methyl esters (FAMEs). The analyses were performed with a gas chromatograph (7890B GC System-G1513A autosampler, Agilent Technologies^®^, Santa Clara, CA, USA) connected to a mass selective detector (5977B MSD, Agilent Technologies^®^). The fractions were injected (1 μL) at 250 °C and separated with a constant helium flow rate (1 mL min^−1^) on an HP-5ms Ultra Inert column (30 m, 0.25 mm, and 0.25 μm, Agilent Technologies^®^), as described in [39]. The run procedure was configured as follows for the MeOH fractions: start at 60 °C for 1 min, increase by 25 °C min^−1^ to 235 °C, hold at 235 °C for 3 min, increase by 3 °C min^−1^ to 242 °C and by 35 °C min^−1^ to 325 °C, end at 60 °C after a 3 min post-run. The CHCl_3_ fractions were run as follows: start at 100 °C for 1 min, increase by 35 °C min^−1^ to 175 °C, by 5 °C min^−1^ to 200 °C, by 8 °C min^−1^ to 215 °C, and by 15 °C min^−1^ to 325 °C, and end at 100°C for 3 min after the post-run. The protocol used was designed for an untargeted metabolomics approach, exploring various fractions (MeOH and CHCl_3_) with different derivatization strategies, rather than for targeted fatty acid analyses.

Data processing and metabolites annotation were performed as described in [39]. For both fractions, the parameters were modified as follows: peak deconvolution (min.peak.width = 1.8), min.peak.height = 1500, noise.threshold = 500, avoid.processing.mz = c (73,149,207), peak alignment (min.spectra.cor = 0.75, max.time.dist = 2, mz.range = 40:600), and missing compoundsrecovery (recMissComp function, minimum number of samples was set at two). The compounds were annotated by comparison with the NIST mass spectra database and by calculating the Kovats retention index [64]. Two csv files were obtained and were used to construct the GC-MS-CHCl_3_ (X2 of 26 *m*/*z* columns) and GC-MS-MeOH (X3 of 42 *m*/*z* columns) matrices.

##### NMR Analyses and Spectral Attributions

The samples were prepared in 600 µL deuterated methanol, CD_3_OD, with 1 mg of dry extract in 5 mm diameter NMR tubes. Proton 1D spectra were obtained at 600 MHz and 298 K using a Bruker Avance III HD spectrometer (Wissembourg, France) equipped with a 5 mm reversed TCI cryoprobe. Proton 1D spectra were obtained at 600 MHz and 298 K using a Bruker Avance III HD spectrometer (Wissembourg, France) equipped with a 5 mm reversed TCI cryoprobe. One-dimensional free induction decays (FIDs) were acquired with a single 90° pulse sequence on 64K data points for 20.0 ppm spectral width, with a 1 s relaxation delay and 256 scan accumulations. To aid in the identification of discriminant metabolites, some samples from each condition were used to acquire 2D TOCSY (DIPSI) and HSQC sequences. The 2D TOCSY (DIPSI) experiment used 2K data points for F2 and 0.25K data points for F1, with a spectral width of 12.0 ppm in both dimensions, an 80 ms spin lock, and 32 scan accumulations. The 2D HSQC experiment used 1K data points for F2 and 0.5K data points for F1, with F2 having a spectral width of 12.0 ppm and F1 having a spectral width of 219 ppm, with 48 scan accumulations.

Data processing was performed as described in [65], with subtle modifications, as follows: baseline model with spline interpolation algorithms, chemical shift calibration (deuterated methanol, CD_3_OD, residual solvent peak at δ_H_ 3.31 ppm), global spectral alignment with the mean spectrum as reference, and further manual alignment to specific regions with zeros as missing fill values. The bins were selected between 9.0 and 0.2 ppm. This produced an X4 matrix used for multivariate statistical analyses. The regions associated with solvent signal are excluded (4.74 to 4.94 ppm water signal and 3.26 to 3.36 ppm methanol residual signal). The final X4 included a line for each sample and 8800 columns for the remaining bins designated with their central chemical shift values. The Human Metabolome Database [66] and Biological Magnetic Resonance Data Bank [67] served as resources for compound identification. To contextualize the preliminary attributions, the discriminant variables were compared with the scarce existing literature on diatom and microalgal ^1^H NMR metabolomics [33,46,68,69,70,71]. The SMART 2.1 online tool was also used as described in [65].

#### 4.2.2. Statistical Analyses

Statistical analyses were carried out using an in-house script for MATLAB^®^ R2023b for macOS (Mathworks, Natick, MA, USA). A qualitative matrix Y indicating the affiliation of each sample to a specific group was constructed. All lines of the matrices were normalized using probabilistic quotient normalization (PQN) [72]. Logarithmic transformation was applied to the X1, X2, and X3 matrices (to allow logarithmic transformations, zeros in the data set were replaced with values of 1000). All four matrices were centered before variable scaling, using Pareto scaling for LC-HRMS^2^ (matrix X1) and unit variance scaling for the two GC-MS mass spectrometry matrices (X2 and X3) and the NMR matrix (X4).

Principal Component Analyses (PCA) and Projection on Latent Structures (PLS) analyses were performed separately with each X matrix.

Prior to multiblock analysis, each block was scaled to unit variance by dividing it by its own variance to ensure that differences in variable count across blocks did not bias the results.

The MBPLS approach is a supervised model that combines MPCA and PLS [73]. The MBPLS model’s results are presented as a biplot, including a score plot and a loading plot. The score plot visualizes the projection of individual samples along the principal components. Each point in the score plot represents a sample, and its coordinates correspond to the scores on the selected components. The loading plot is constructed for each block and for each component. Each point in the loading plot corresponds to a variable’s projection onto the subspace defined by the displayed component(s). The coordinates of these points correspond to the weight matrices of the models (W^MBPLS^).

Only variables with loading values that fall within the upper quarter of the range of the absolute values are displayed and were considered as discriminant variables. The coordinates of these variables are uniformly scaled to compensate for block normalization effects and ensure comparability. This scaling preserves the angle between the variable’s projection and the component axes. Similarly, the scores of the differences are scaled to maintain the angle of the individual’s projection with respect to the component axes.

The statistical performance of the models (PLS or MBPLS) was evaluated using the R^2^Y fit parameter, the Q^2^Y cross-validated coefficient of determination parameter, and the AUROC (Area Under the Receiver Operating Characteristic Curve). R^2^Y represents the proportion of Y variance that is explained by the model. Q^2^Y (calculated using the ‘leave-one-out’ cross-validation method) measures the model’s predictability in terms of how well the model should predict Y in new data. AUROC evaluates predictability in terms of the model’s ability to correctly rank or separate classes.

## 5. Conclusions

The metabolomics strategy described in this study allowed us to classify culture conditions through an integrative approach. The first classification obtained showed different metabolomic profiles of planktonic culture compared to adherent cultures, whatever the nature of the support. In addition, the second and third components of the statistical model allowed us to classify the three adherent cultures according to their physicochemical properties. Notably, lipid and sugar metabolism modulations were observed under adherent conditions when compared to planktonic cultures. CG cultures, grown on both hard and hydrophilic supports, exhibited a metabolic shift of PUFA, SQDG, sterol, and amino acid profiles when compared to hydrophobic supports. Diatom growth on CPT, a hard surface, and CPDMS, a soft surface, both hydrophobic, exhibited changes in terpenoids, carbohydrates, and lipid metabolism. To further elucidate the molecular mechanisms underlying diatom adhesion, this omics method using metabolomics should be integrated with transcriptomic studies to identify overexpressed and/or downregulated genes involved in adhesion. We show here that metabolomics contributes to identifying phenotypic markers that will be crucial to better understand their mechanisms and roles in diatom adhesion and the biofouling process. Further studies including transcriptomic or genetic studies are required in order to validate this first metabolomic study on the adhesion process of the diatom *P. tricornutum.*

## Figures and Tables

**Table 1:** Discriminant compounds in the LC-HRMS^2^ analyses on T_1_, T_2_, and T_3_ axes.

Comp.	RT	*m*/*z* (Adduct)	[Adduct], Compound’s Molecular Formula	Compound’s Name	Superclass/Natural Product Class	Err. ppm	Sirius Score	T_1_: Increased in
LC16	9.395	111.0437	[M + H]^+^, C_6_H_6_O_2_	Catechol ^(a) (c)^	Organic oxygen compounds/ Phenolic acids (C6-C1)	−1.3	100.00%	CP
LC19	3.619	115.0389	[M-H_2_O + H]^+^, C_5_H_8_O_4_	N.A.	Organic acids and derivatives/ Dicarboxylic acids	−0.5	100.00%	CG/CPT/CPDMS
LC41	3.572	133.0494	[M-H_2_O + H]^+^, C_5_H_10_O_5_	Ribose ^(a) (c)^	Carboxylic acids/Saccharides	−0.7	100.00%	CG/CPT/CPDMS
LC132	11.582	181.1221	[M + H]^+^, C_11_H_16_O_2_	Dihydroactinidiolide ^(a) (b) (c) (d)^	Lipids and lipid-like molecules/ Terpenoids	−2.7	100.00%	CP
LC303	0.943	249.0377	[M + H_2_O + K]^+^, C_7_H_12_O_6_	Quinic acid ^(a) (c) (d)^	Organic oxygen compounds/Cyclitols	1.6	100.00%	CP
LC650	0.967	395.1156	[M + Na]^+^, C_13_H_24_O_12_	N.A.	Organic oxygen compounds/ Disaccharides	0.7	100.00%	CP
LC695	21.422	433.3304	[M + H]^+^, C_27_H_44_O_4_	N.A.	Lipids and lipid-like molecules/ Terpenoids	3.8	99.78%	CP
LC836	13.488	560.3786	[M + H]^+^, C_29_H_53_NO_9_	DGTA/S 5:1;O2/14:0 ^(e)^	Organic acids and derivatives/ Glycerolipids	1.2	100.00%	CG/CPT/CPDMS
LC855	13.643	574.3941	[M + H]^+^, C_30_H_55_NO_9_	DGTA/S 6:1;O2/14:0 ^(e)^	Organic acids and derivatives/ Glycerolipids	0.5	99.90%	CG/CPT/CPDMS
LC872	13.916	588.4099	[M + H]^+^, C_31_H_57_NO_9_	DGTA/S 7:1;O2/14:0 ^(e)^	Organic acids and derivatives/ Glycerolipids	0.3	99.95%	CG/CPT/CPDMS
LC873	14.567	588.4100	[M + H]^+^, C_31_H_57_NO_9_	DGTA/S 5:1;O2/16:0 ^(e)^	Organic acids and derivatives/ Glycerolipids	−1.4	99.95%	CG/CPT/CPDMS
LC891	14.106	602.4255	[M + H]^+^, C_32_H_59_NO_9_	DGTA/S 8:1;O2/14:0 ^(e)^	Organic acids and derivatives/ Glycerolipids	−0.1	98.14%	CG/CPT/CPDMS
LC912	14.434	616.4411	[M + H]^+^, C_33_H_61_NO_9_	DGTA/S 9:1;O2/14:0 ^(e)^	Organic acids and derivatives/ Glycerolipids	0.3	67.18%	CG/CPT/CPDMS
LC944	15.495	644.4726	[M + H]^+^, C_35_H_65_NO_9_	DGTA/S 9:1;O2/16:0 ^(e)^	Organic acids and derivatives/ Glycerolipids	0.1	94.37%	CG/CPT/CPDMS
LC1011	18.141	724.5712	[M + H]^+^, C_42_H_77_NO_8_	DGTA/S 18:2;O1/14:0 ^(e)^	Organic acids and derivatives/ Glycerolipids	1.5	97.89%	CG/CPT/CPDMS
LC1024	19.204	752.6032	[M + H]^+^, C_44_H_81_NO_8_	DGTA/S 18:2;O1/16:0 ^(e)^	Organic acids and derivatives/ Glycerolipids	1.5	99.46%	CG/CPT/CPDMS
**Comp.**	**RT**	***m*/*z* (Adduct)**	**[Adduct], Compound’s Molecular formula**	**Compound’s Name**	**Superclass/Natural Product Class**	**Err. ppm**	**Sirius Score**	**T_2_: Increased in**
LC718	7.663	454.1831	[M + H]^+^, C_18_H_32_NO_12_	DGTA/S 5:1;O2/3:1;O3 ^(e)^	Organic acids and derivatives/ Glycerolipids	23.50	N.A.	CG
LC729	6.495	464.2121	[M + H]^+^, C_20_H_34_NO_11_	DGTA/S 5:1;O2/5:1;O2 ^(e)^	Organic acids and derivatives/ Glycerolipids	−0.04	86.99%	CG
LC746	6.94	478.2278	[M + H]^+^, C_21_H_35_NO_11_	DGTA/S 5:1;O2/6:1,O2 ^(e)^	Organic acids and derivatives/ Glycerolipids	1.28	40.19%	CG
LC762	7.553	492.2435	[M + H]^+^, C_22_H_37_NO_11_	DGTA/S 5:1;O2/7:1;O2 ^(e)^	Organic acids and derivatives/ Glycerolipids	−1.14	89.30%	CG
LC768	9.304	496.2300	[M + H]^+^, C_21_H_37_NO_12_	DGTA/S 5:1;O2/6:0,O3 ^(e)^	Organic acids and derivatives/ Glycerolipids	19.67	N.A.	CG
LC790	9.823	510.2456	[M + H]^+^, C_22_H_39_NO_12_	DGTA/S 5:1;O2/7:0;O3 ^(e)^	Organic acids and derivatives/ Glycerolipids	14.00	N.A.	CG
LC812	8.925	534.2902	[M + H]^+^, C_25_H_43_NO_11_	DGTA/S 6:1;O2/9:1;O2 ^(e)^	Organic acids and derivatives/ Glycerolipids	1.50	79.50%	CG
LC885	11.867	600.3733	[M + H], C_31_H_53_NO_10_	DGTA/S 5:1;O2/16:2;O ^(e)^	Organic acids and derivatives/ Glycerolipids	0.10	94.66%	CG
**Comp.**	**RT**	***m*/*z* (Adduct)**	**[Adduct], Compound’s Molecular formula**	**Compound’s** **Name**	**Superclass/Natural Product Class**	**Err. ppm**	**Sirius Score**	**T_3_: Increased in**
LC52	2.648	141.1385	[M + H]^+^, C_8_H_16_N_2_	N.A.	N.A.	0.9	100.00%	CPT
LC73	8.993	149.0706	[M + H]^+^, C_8_H_8_N_2_O	N.A.	Organic oxygen compounds/ Alkaloids	2.3	100.00%	CPT
LC241	12.962	225.1957	[M + H]^+^, C_13_H_24_N_2_O	N.A.	Organic oxygen compounds/ Alkaloids	−0.6	94.37%	CPT
LC315	14.774	253.1908	[M + H]^+^, C_14_H_24_N_2_O_2_	N.A.	Organic oxygen compounds/ Alkaloids	1.8	97.89%	CPT
LC1039	22.596	791.6748	[M + H_2_O + H]^+^, C_49_H_88_O_6_	TG 14:0/16:0/16:2 ^(e)^	Lipids and lipid-liked molecules/ Fatty acids	2.1	92.21%	CPDMS

Annotation of error ppm was performed using Data Analyses 4.4, Sirius score was annotated using the version 7.2, Rt for retention time, annotation from Sirius ^(a)^, GNPS ^(b)^, tima-R ^(c)^, and Metaboscape ^(d)^, manual annotation of MS2 ^(e)^.

**Table 2:** Discriminant compounds in the CHCl_3_ fractions (after data analysis and filtering) on the T_1_, T_2_, and T_3_ axes.

Comp.	Molecular Name	Pathway	Raw Formula	Match NIST	CAS Number	Exp. RI	Est. RI	Litt. RI	T_1_: Increased in
C4	Tetradecanoic acid (C14:0)	Fatty acids	C_14_H_28_O_2_	956	544-63-8	1731	-	1725	CG/CPT/CPDMS
C10	(7*Z*,10*Z*)-Hexadecadienoic acid (C16:2n-6)	Fatty acids	C_16_H_28_O_2_	844	28290-73-5	1894	1894	-	CP
C18	Phytol	Terpenoids	C_20_H_40_O	723	150-86-7	2071	-	2114	CP
C24	9,10,12-Trihydroxyoctadecanoic acid (C18:0;O3)	Fatty acids	C_18_H_36_O_5_	651 *	25027-95-6	2282	-	-	CP
**Comp.**	**Molecular Name**	**Pathway**	**Raw Formula**	**Match NIST**	**CAS Number**	**Exp. RI**	**Est. RI**	**Litt. RI**	**T_2_: Increased in**
C9	2,6,10,15-Tetramethylheptadecane	Alkane	C_21_H_44_	730	54833-48-6	1889	-	1889	CP/CPT/CPDMS
C19	(6*Z*,9*Z*,12*Z*,15*Z*)-Octadecatetraenoic acid (C18:4n-3)	Fatty acids	C_18_H_28_O_2_	925	20290-75-9	2094	-	2088	CG
C26	4,7,10,13,16,19-Docosahexaenoic acid (C22:6n-3)	Fatty acids	C_22_H_32_O_2_	819	2091-24-9	2456	-	2471	CG
**Comp.**	**Molecular Name**	**Pathway**	**Raw Formula**	**Match NIST**	**CAS Number**	**Exp. RI**	**Est. RI**	**Litt. RI**	**T_3_: Increased in**
C8	2-Methyloctadecane	Alkane	C_19_H_40_	810	1560-88-9	1856	-	1863	CPT
C12	(9*Z*)-Hexadecenoic acid (C16:1n-7)	Fatty acids	C_16_H_30_O_2_	960	10030-73-6	1913	-	1904	CPT
C16	Isophytol, acetate	Terpenoids	C_22_H_42_O_2_	718	58425-36-8	2043	-	2064	CPT

Annotation was performed with NIST 2017 and by comparison with a standard mixture for fatty acid methyl esters (comp. compound; RI, Van den Dool and Kratz Retention Index; Exp, experimental; Est, estimated; lit. literature). * Indicates agreement with the NIST 2017 library < 700. N.A., Not assigned.

**Table 3:** Discriminant compounds in the MeOH fractions (after data analysis and filtering) on theT_1_, T_2_, and T_3_ axes.

Comp.	Molecular Name	Pathway	Raw Formula	Match NIST	CAS Number	Exp. RI	Est. RI	Litt. RI	T_1_: Increased in
M8	D-Ribofuranose (isomer 2)	Carbohydrates	C_5_H_10_O_5_	836	613-83-2	1629	-	1641	CG/CPT/CPDMS
M10	D-Ribose	Carbohydrates	C_5_H_10_O_5_	868	10257-32-6	1677	1651	-	CG/CPT/CPDMS
M16	Unknown sugar	Carbohydrates	N.A.	N.A.	N.A.	1784	-	-	CP
M29	*Myo*-Inositol	Carbohydrates	C_6_H_12_O_6_	762	551-72-4	2068	2194	-	CG/CPT/CPDMS
M33	(9*Z*)-Octadecenoic acid (C18:1n-9)	Fatty acids	C_18_H_34_O_2_	780	112-80-1	2166	-	2141	CG/CPT/CPDMS
M42	D-Cellobiose	Carbohydrates	C_12_H_22_O_11_	711	528-50-7	-	-	2762	CP
**Comp.**	**Molecular Name**	**Pathway**	**Raw Formula**	**Match NIST**	**CAS Number**	**Exp. RI**	**Est. RI**	**Litt. RI**	**T_2_: Increased in**
M3	Glycerol	Polyol	C_3_H_8_O_3_	894	56-81-5	1221	1066	1247	CP/CPT/CPDMS
M28	*N*-Acetyl-D-Glucosamine (isomer 1)	Carbohydrates	C_8_H_15_NO_6_	637	7512-17-6	2061	-	2068	CP/CPT/CPDMS
**Comp.**	**Molecular Name**	**Pathway**	**Raw Formula**	**Match NIST**	**CAS Number**	**Exp. RI**	**Est. RI**	**Litt. RI**	**T_3_: Increased in**
M26	D-Glucuronic acid	Carbohydrates	C_6_H_10_O_7_	795	528-16-5	2024	-	2012	CPT
M36	D-Glucose	Carbohydrates	C_6_H_12_O_6_	841	2280-44-6	2193	2173	-	CPT

Annotation was performed with NIST 2017 and by comparison with a standard mixture for fatty acid methyl esters (comp. compound; RI, Van den Dool and Kratz Retention Index; Exp, experimental; Est, estimated; lit. literature). * Indicates agreement with NIST 2017 library < 700. N.A. assigned.

**Table 4:** Discriminant variables on the T_1_, T_2_, T_3_ axes from the NMR block relevant for the assignment of the main resonances of P. tricornutum (600 MHz, in CD_3_OD).

Discriminant Chemical Shift on Axis 1 (δ in ppm) and Multiplicity	Attribution	Annotation Confidence	T_1_: Increased in
0.90, t	Fatty acids CH_3_	4	CG/CPT/CPDMS
1.12, t	N.A.	0	CG/CPT/CPDMS
1.30, bs	Fatty acids CH_2_	4	CG/CPT/CPDMS
1.60, t	Fatty acids CH_2_ β-ester	4	CG/CPT/CPDMS
2.35, t	Fatty acids CH_2_ α-ester	4	CG/CPT/CPDMS
2.90, dd	2,3-dihydroxypropane-1-sulfonate (DHPS)	4	CP
2.95, s	Dimethylsulfoniopropionate (DMSP)	4	CP
3.04, dd	2,3-dihydroxypropane-1-sulfonate (DHPS)	4	CP
3.21, s	Choline	4	CP
3.40, t	Dimethylsulfoniopropionate (DMSP)	4	CP
3.44,	Carbohydrates (glucose/galactose)	2	CP
3.54, tt	Glycerophospholipids/Glycerolipids	2	CG/CPT/CPDMS
3.62, dd	2,3-dihydroxypropane-1-sulfonate (DHPS)	4	CP
3.65, bs	Glycerol	4	CG/CPT/CPDMS
3.75, d	Carbohydrates (glucose/galactose)	2	CP
3.82, m	Glycerophospholipids/Glycerolipids	2	CG/CPT/CPDMS
3.85, bs	Carbohydrates (glucose/galactose)	2	CP
3.94, m	Carbohydrates (glucose/galactose)	2	CP
4.01, dd	Glycerophospholipids/Glycerolipids	2	CG/CPT/CPDMS
4.10, dd	Glycerophospholipids/Glycerolipids	2	CG/CPT/CPDMS
8.40, s	Formate	4	CG/CPT/CPDMS
**Discriminant Chemical Shift on Axis 2 (δ in ppm) and ** **Multiplicity**	**Attribution**	**Annotation Confidence**	**T_2_: Increased in**
0.74, t	Sterols	3	CG
1.01, d	Isoleucine	4	CG
1.03, d	Valine	4	CG
1.45, d	Lactate	4	CPT/CPDMS/CP
1.60, t	Fatty acids CH_2_ β ester	3	CG
2.32, m	Proline	4	CG
2.60, t	Fatty acids CH_2_ α ester	3	CG
2.82, s	N.A.	0	CG
3.21, s	Choline	4	CG
3.65, bs	Glycerol	4	CPT/CPDMS/CP
4.12, dd	Sulphoquinovosyldiacylglycerols (SQDGs)	4	CG
4.19, dd	Sulphoquinovosyldiacylglycerols (SQDGs)	4	CG
6.10, dd	N.A.	0	CG
6.92, dd	N.A.	0	CG
**Discriminant Chemical Shift on Axis 3 (δ in ppm) and** **Multiplicity**	**Attribution**	**Annotation Confidence**	**T_3_: Increased in**
0.90, t	Fatty acids CH_3_	3	CPT
1.30, bs	Fatty acids CH_2_	3	CPT
1.60, t	Fatty acids CH_2_ β ester	3	CPT
2.35, t	Fatty acids CH_2_ α ester	3	CPT
4.10, dd	Glycerophospholipids/Glycerolipids	1	CPT

Annotation confidence assignments are as follows: 0 = N.A. (not assigned); 1 = putative attribution with functional group information; 2 = partially matched to HSQC chemical shift information in the databases or literature; 3 = fully matched to HSQC chemical shift; 4 = fully matched to HSQC chemical shift and validated by HSQC-TOCSY.

## Data Availability

The datasets supporting this article will be made available on request.

## Supplementary Materials

The following supporting information can be downloaded at: https://www.mdpi.com/article/10.3390/md23080314/s1, Figure S1: Feature-based molecular networking (https://gnps.ucsd.edu/ProteoSAFe/status.jsp?task=3d688a78ced74bef860bb1ce6fbcf402, accessed on 24 May 2023); Figure S2: Collapsed ion molecular networking from FBMN annotated with NPClassifier (https://gnps.ucsd.edu/ProteoSAFe/status.jsp?task=3d688a78ced74bef860bb1ce6fbcf402, accessed on 24 May 2023); Figure S3: Collapsed ion molecular networking from FBMN annotated with Classifire (https://gnps.ucsd.edu/ProteoSAFe/status.jsp?task=3d688a78ced74bef860bb1ce6fbcf402, accessed on 24 May 2023); Figure S4: MS/MS fragmentation patterns of lipids of interest discriminant on T_1_ axis; Figure S5: Discriminant compounds on T_1_ axis and their clusters; when available, the planar structure of the molecules is shown, taking into account the annotations from the different pipelines used (GNPS, Sirius, tima-R, Metaboscape, Manual annotation MS2). Chemical family corresponding data from ConCISE and refinement from Sirius. (CG = glass, CPT = polystyrene, CPDMS = polydimethylsiloxane, CP = planktonic); Figure S6: Discriminant compounds on T_2_ axis and their clusters; when available, the planar structure of the molecules is shown, taking into account the annotation from the different pipelines used (GNPS, Sirius, tima-R, Metaboscape, Manual annotation MS2). Chemical family corresponding data from ConCISE and refinement from Sirius. (CG = glass, CPT = polystyrene, CPDMS = polydimethylsiloxane, CP = planktonic); Figure S7: Discriminant compounds on T_3_ axis and their clusters; when available, the planar structure of the molecules is shown, taking into account the annotation from the different pipelines used (GNPS, Sirius, tima-R, Metaboscape, Manual annotation MS2). Chemical family corresponding data from ConCISE and refinement from Sirius. (CG = glass, CPT = polystyrene, CPDMS = polydimethylsiloxane, CP = planktonic); Figure S8: TOCSY-NMR spectrum of the X3.0 planktonic culture (600 MHz, CD_3_OD); Figure S9: HSQC-ED-NMR spectrum of the X3.0 planktonic culture (600 MHz, CD_3_OD); Figure S10: TOCSY-NMR spectrum of the C1.0 CPDMS adherent culture (600 MHz, CD_3_OD); Figure S11: HSQC-ED-NMR spectrum of the C1.0 CPDMS adherent culture (600 MHz, CD_3_OD); Table S1: Discriminants compounds from the LC-HRMS^2^, GC/MS, and NMR analyses on T_1_ axis; Table S2: Discriminants compounds from the LC-HRMS^2^, GC/MS, and NMR analyses on T_2_ axis; Table S3: Discriminants compounds from the LC-HRMS^2^, GC/MS, and NMR analyses on T_3_ axis.
