# Supplementary material for: Multiblock Metabolomics Responses of the Diatom Phaeodactylum tricornutum Under Benthic and Planktonic Culture Conditions
Source: Mar Drugs. 2025 Jul 31;23(8):314. doi: 10.3390/md23080314 (PMC12387633; doi:10.3390/md23080314)
Supplement: Supplementary file 1 [file marinedrugs-23-00314-s001.zip › marinedrugs-3707601-supplementary.pdf]

Supplementary materials

Multiblock Metabolomics Responses of the Diatom *Phaeodactylum tricornutum* Under Benthic and Planktonic Culture Conditions

Andrea Castaldi [1,2], Mohamed N. Triba [3], Laurence Le Moyec [1,4], Cedric Hubas [5], Gaël Le Pennec [2,*], Marie-Lise Bourguet-Kondracki [1,*]

1 Molécules de Communication et Adaptation des Microorganismes, UMR 7245 CNRS, Muséum National d'Histoire Naturelle, 57 rue Cuvier (CP54), 75005 Paris, France.
2 Laboratoire de Biotechnologie et Chimie Marines, Université Bretagne Sud, EMR CNRS 6076, IUEM, 56100 Lorient, France.
3 CB3S (Chimie Bioorganique, Biophysique, Biomatériaux pour la Santé)—UMR 7244 CNRS, Université Paris 13, 74 rue Marcel Cachin, 93017 Bobigny Cedex, France.
4 Université d'Evry Val d'Essonne - Paris Saclay, Boulevard F. Mitterrand, 91025 Evry, France.
5 Muséum National d'Histoire Naturelle, UMR BOREA, MNHN-CNRS-UCN-UPMC-IRD-UA, Station Marine de Concarneau, Concarneau, 75005 Paris, France.
* Correspondence: gael.le-pennec@univ-ubs.fr (G.L.P.); marie-lise.bourguet@mnhn.fr (M.-L.B.-K.)

Figure S1: Feature based molecular networking (https://gnps.ucsd.edu/ProteoSAFe/status.jsp?task=3d688a78ced74bef860bb1ce6fbcf402, accessed on 24 May 2023)

Figure S2: Collapsed ion molecular networking from FBMN annotated with NPClassifier (https://gnps.ucsd.edu/ProteoSAFe/status.jsp?task=3d688a78ced74bef860bb1ce6fbcf402, accessed on 24 May 2023)

Figure S3: Collapsed ion molecular networking from FBMN annotated with Classifire (https://gnps.ucsd.edu/ProteoSAFe/status.jsp?task=3d688a78ced74bef860bb1ce6fbcf402, accessed on 24 May 2023)

Figure S4: MS/MS fragmentation patterns of lipids of interest discriminant on T_1 axis.

Figure S5: Discriminant compounds on T_1 axis and their clusters; when available, the planar structure of the molecules is shown, taking in account the annotation from the different pipelines used (GNPS, Sirius, tima-R, Metaboscape, Manual annotation MS2). Chemical family corresponding data from ConCISE and refinement from Sirius (CG = glass, CPT = polystyrene, CPDMS = polydimethylsiloxane, CP = planktonic).

Figure S6: Discriminant compounds on T_2 axis and their clusters; when available, the planar structure of the molecules is shown, taking into account the annotation from the different pipelines used (GNPS, Sirius, tima-R, Metaboscape, Manual annotation MS2). Chemical family corresponding data from ConCISE and refinement from Sirius. (CG = glass, CPT = polystyrene, CPDMS = polydimethylsiloxane, CP = planktonic).

Figure S7: Discriminant compounds on T_3 axis and their clusters; when available, the planar structure of the molecules is shown, taking into account the annotation from the different pipelines used (GNPS, Sirius, tima-R, Metaboscape, Manual annotation MS2). Chemical family corresponding data from ConCISE and refinement from Sirius. (CG = glass, CPT = polystyrene, CPDMS = polydimethylsiloxane, CP = planktonic).

Figure S8: TOCSY-NMR spectrum of the X3.0 planktonic culture (600 MHz, CD_3OD).

Figure S9: HSQC-ED-NMR spectrum of the X3.0 planktonic culture (600 MHz, CD_3OD).

Figure S10: TOCSY-NMR spectrum of the C1.0 CPDMS adherent culture (600 MHz, CD_3OD).

Figure S11: HSQC-ED-NMR spectrum of the C1.0 CPDMS adherent culture (600 MHz, CD_3OD).

Table S1: Discriminants compounds from the LC-HRMS^2, GC/MS, and NMR analyses on T_1 axis.

Table S2: Discriminants compounds from the LC-HRMS^2, GC/MS, and NMR analyses on T_2 axis.

Table S3: Discriminants compounds from the LC-HRMS^2, GC/MS, and NMR analyses on T_3

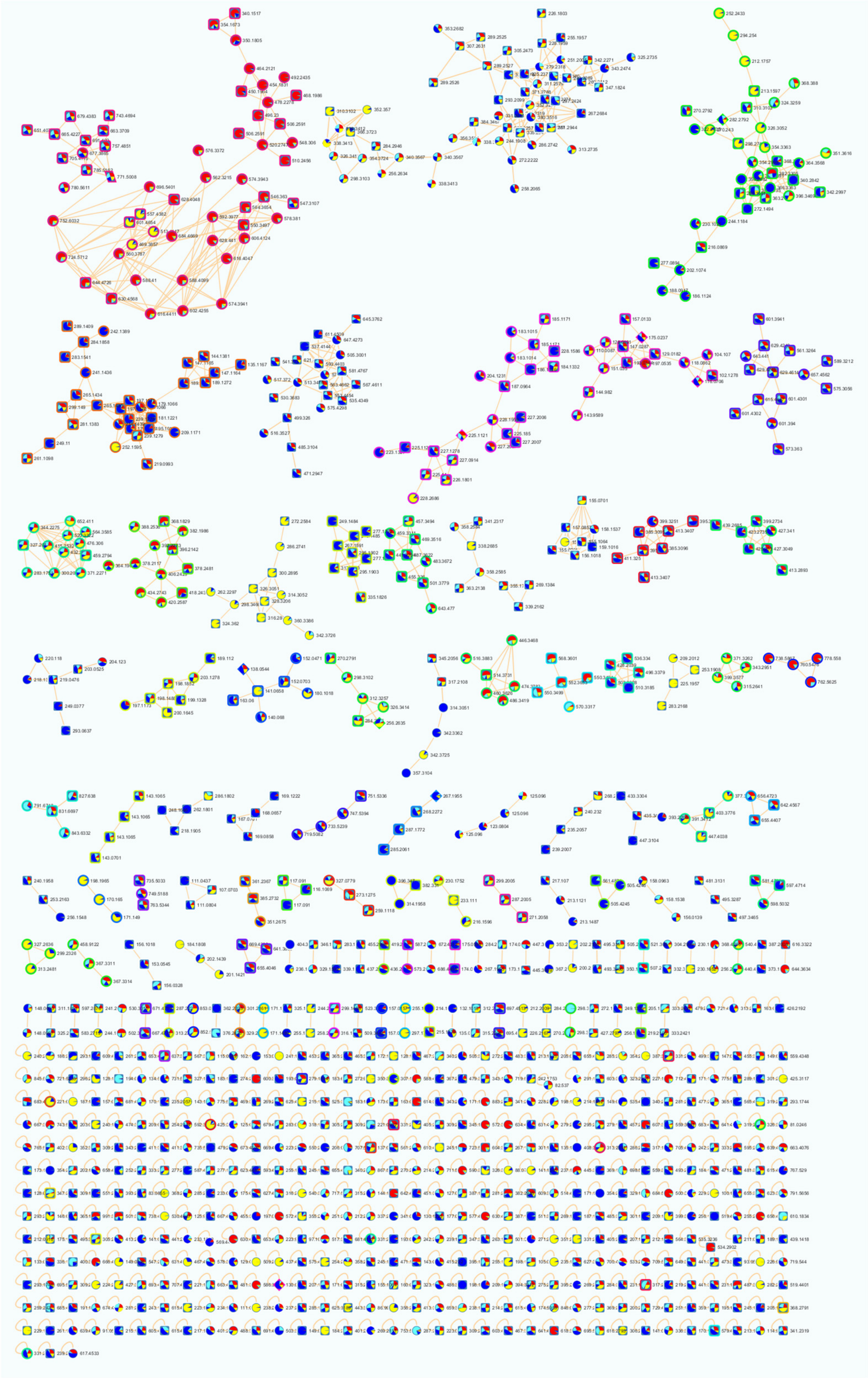

Figure S1: Feature based molecular networking (https://gnps.ucsd.edu/ProteoSAFe/status.jsp?task=3d688a78ced74bef860bb1ce6fbcf402, accessed on 24 May 2023)

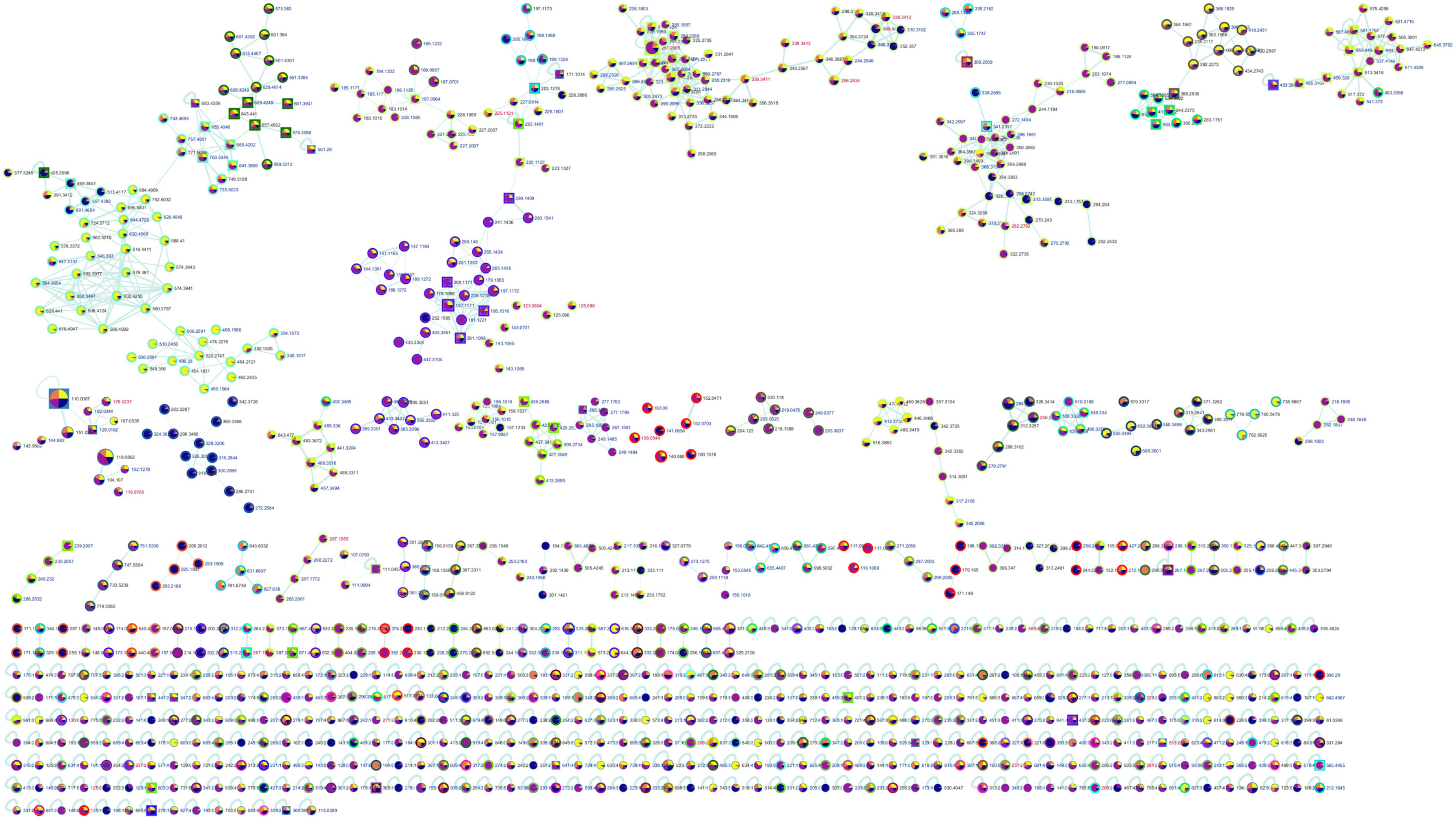

Figure S2: Collapsed ion molecular networking from FBMN annotated with NPClassifier (https://gnps.ucsd.edu/ProteoSAFe/status.jsp?task=3d688a78ced74bef860bb1ce6fbcf402, accessed on 24 May 2023);

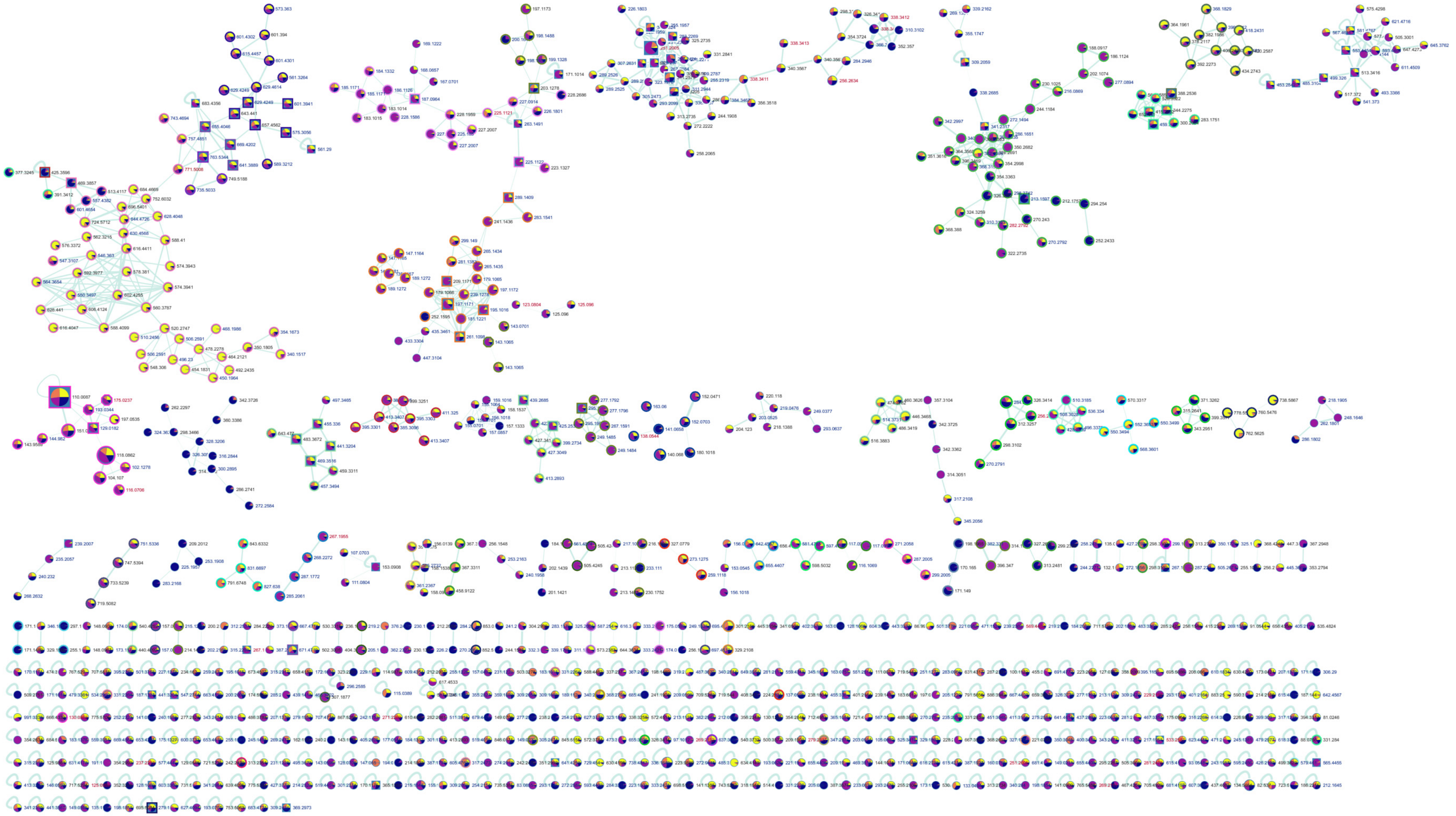

Figure S3: Collapsed ion molecular networking from FBMN annotated with Classifire (https://gnps.ucsd.edu/ProteoSAFe/status.jsp?task=3d688a78ced74bef860bb1ce6fbcf402, accessed on 24 May 2023);

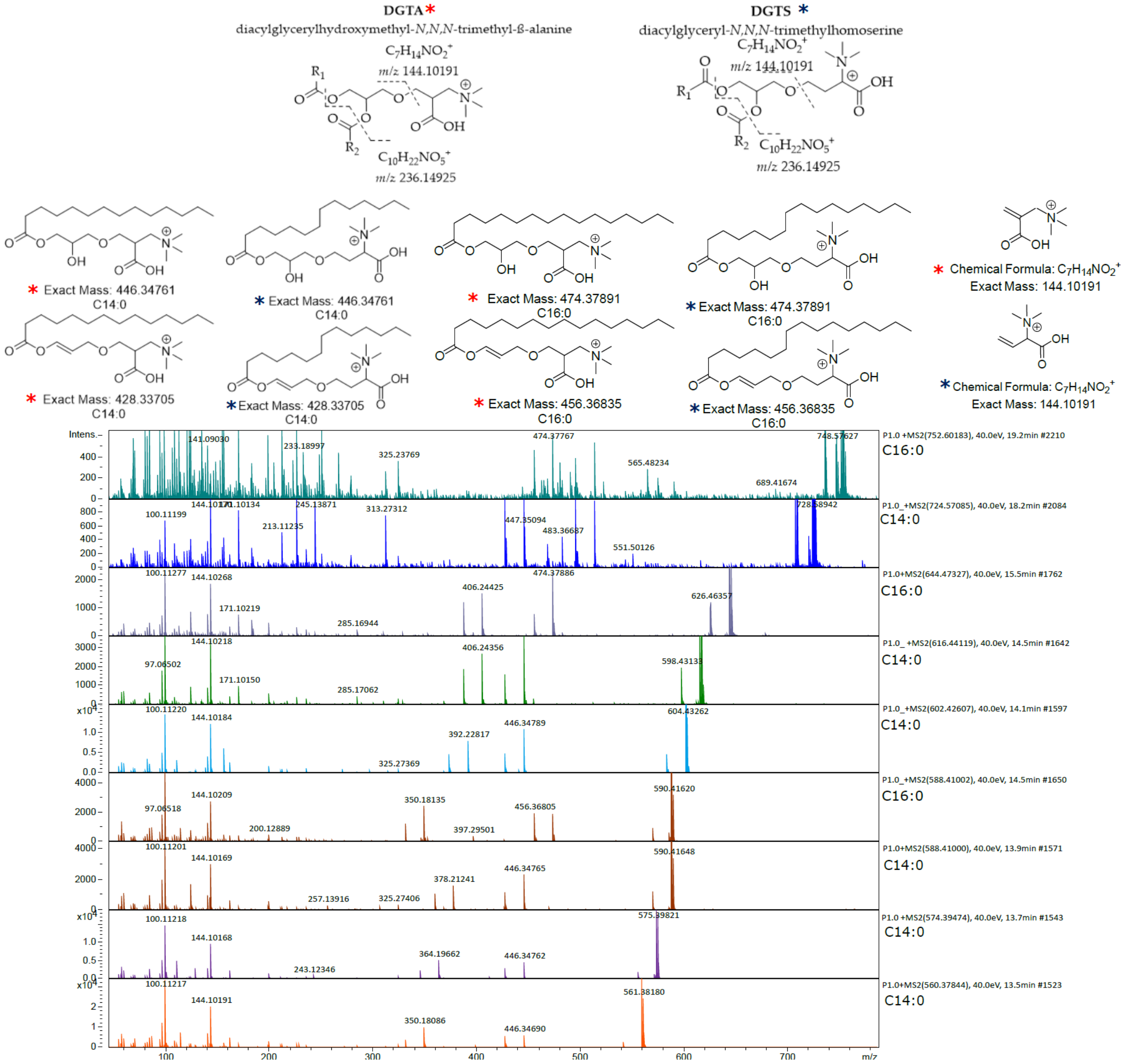

Figure S4: MS/MS fragmentation patterns of lipids of interest discriminant on W^{MBPLS} T$_1$.

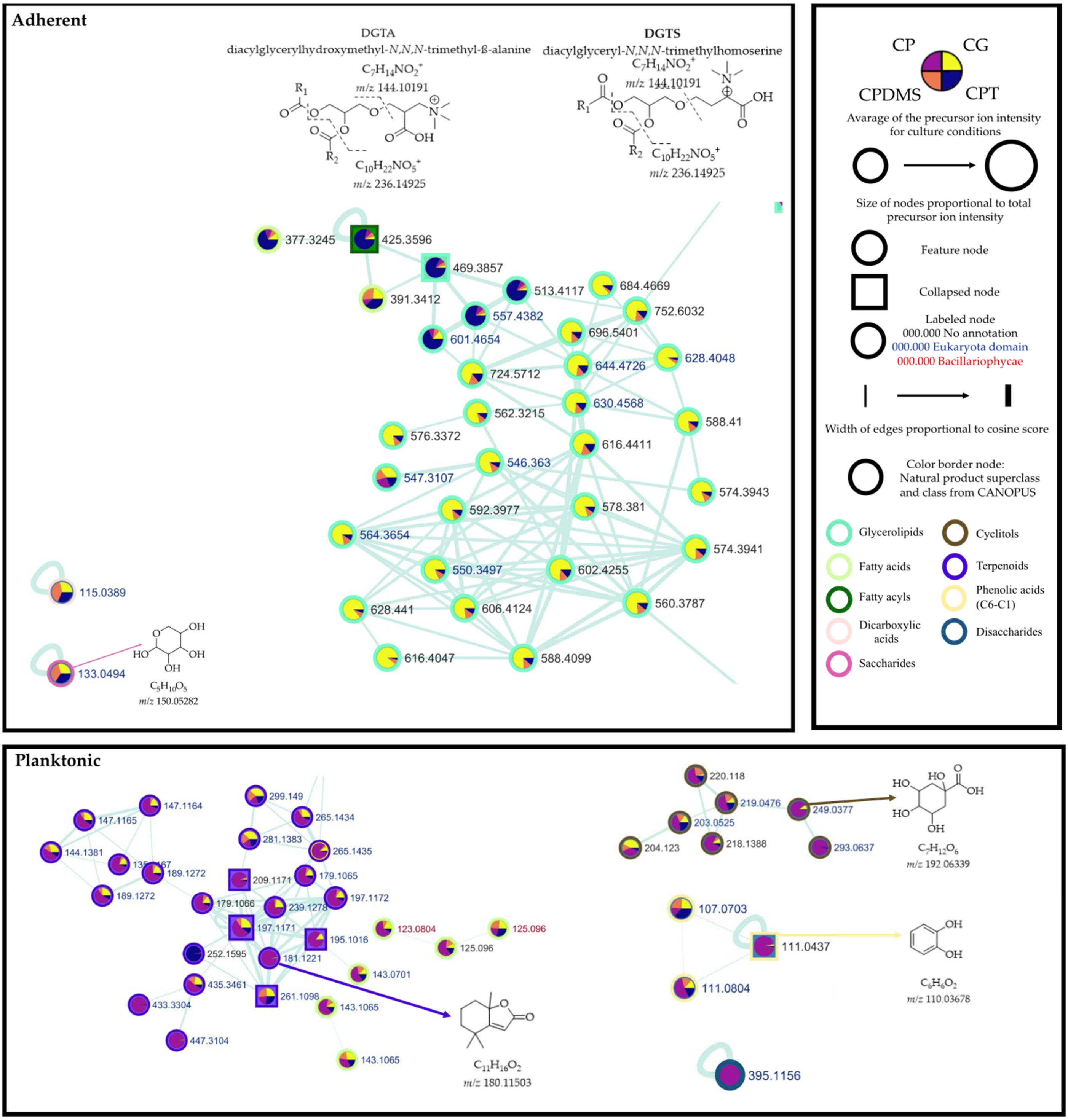

Figure S5: Discriminant compounds on W^{MBPLS} T_1 and their clusters; when available, the planar structure of the molecules is shown, taking into account the annotations from the different pipelines used (GNPS, Sirius, tima-R, Metaboscape, Manual annotation MS2). Chemical family corresponding data from ConCISE and refinement from Sirius. (CG = glass, CPT = polystyrene, CPDMS = polydimethylsiloxane, CP = planktonic);

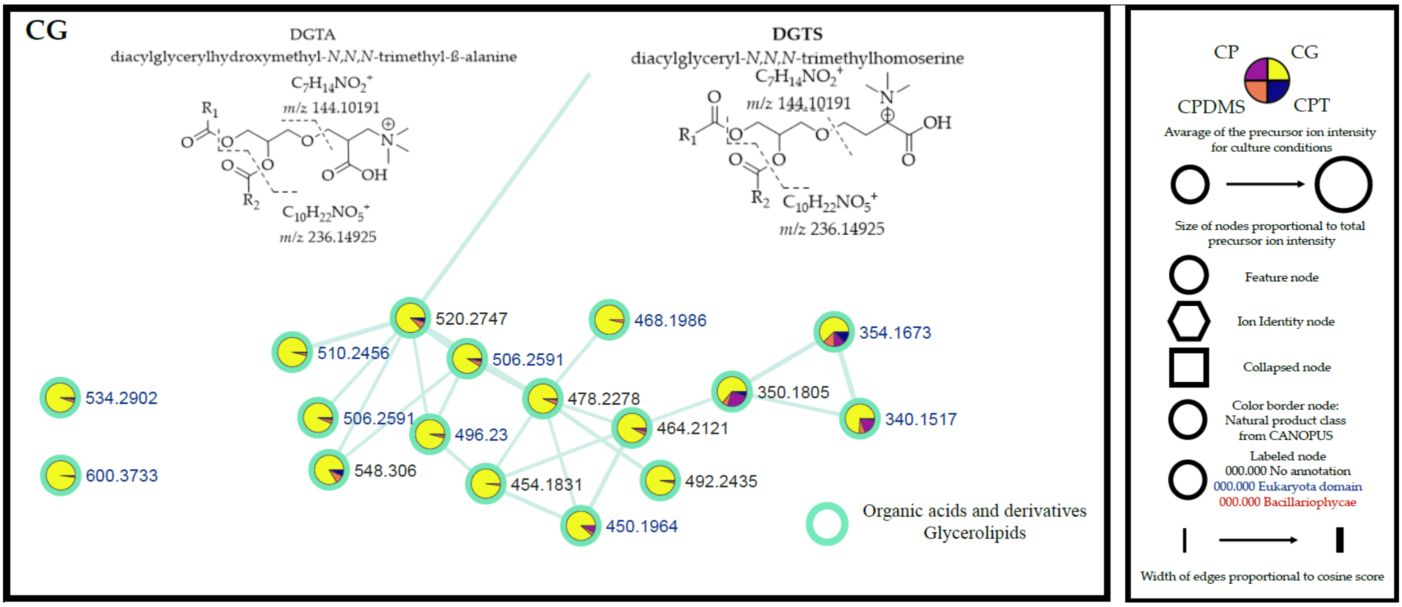

Figure S6: Discriminant compounds on W^{MBPLS} T_2 and their clusters; when available, the planar structure of the molecules is shown, taking into account the annotation from the different pipelines used (GNPS, Sirius, tima-R, Metaboscape, Manual annotation MS2). Chemical family corresponding data from ConCISE and refinement from Sirius. (CG = glass, CPT = polystyrene, CPDMS = polydimethylsiloxane, CP = planktonic).

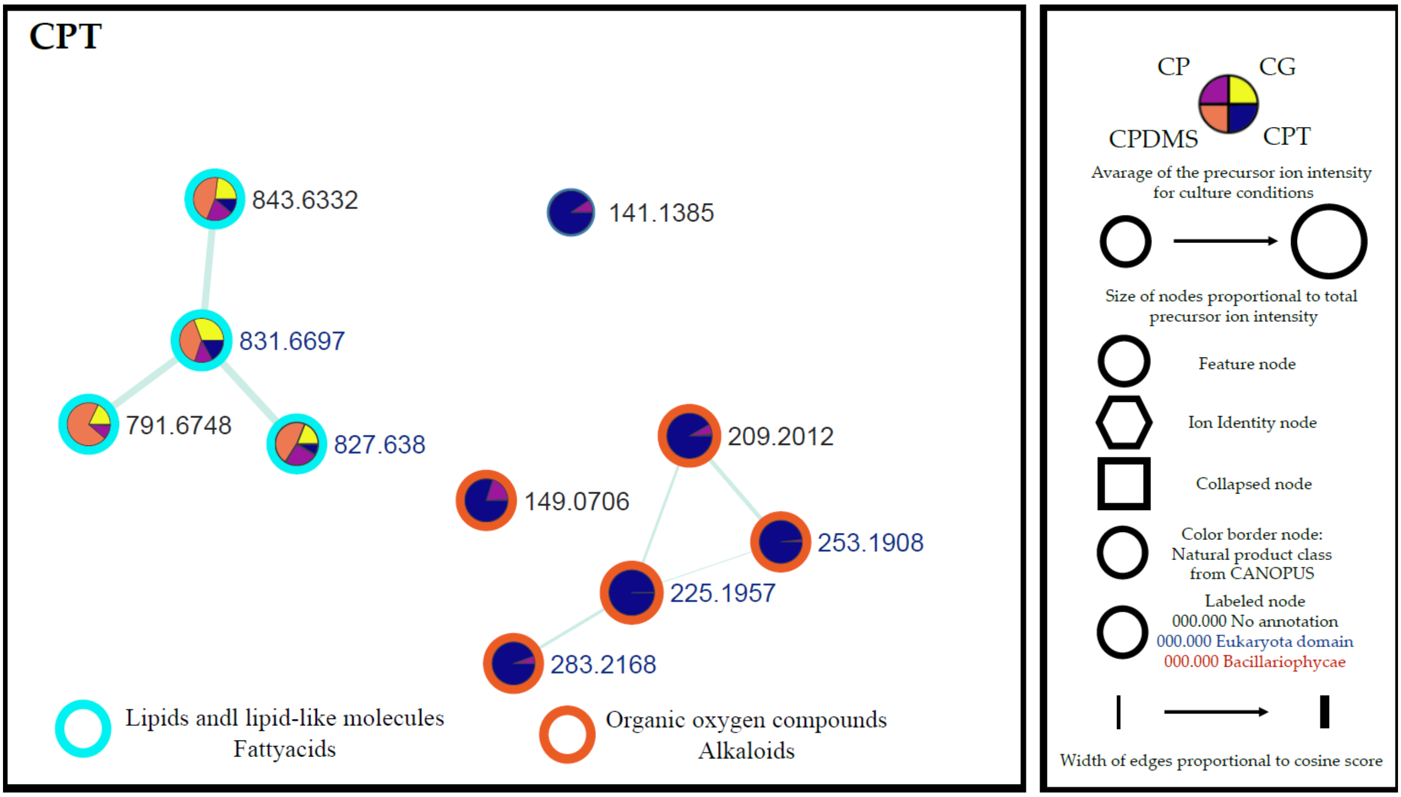

Figure S7: Discriminant compounds on W^{MBPLS} T_3 and their clusters; when available, the planar structure of the molecules is shown, taking into account the annotation from the different pipelines used (GNPS, Sirius, tima-R, Metaboscape, Manual annotation MS2). Chemical family corresponding data from ConCISE and refinement from Sirius. (CG = glass, CPT = polystyrene, CPDMS = polydimethylsiloxane, CP = planktonic).

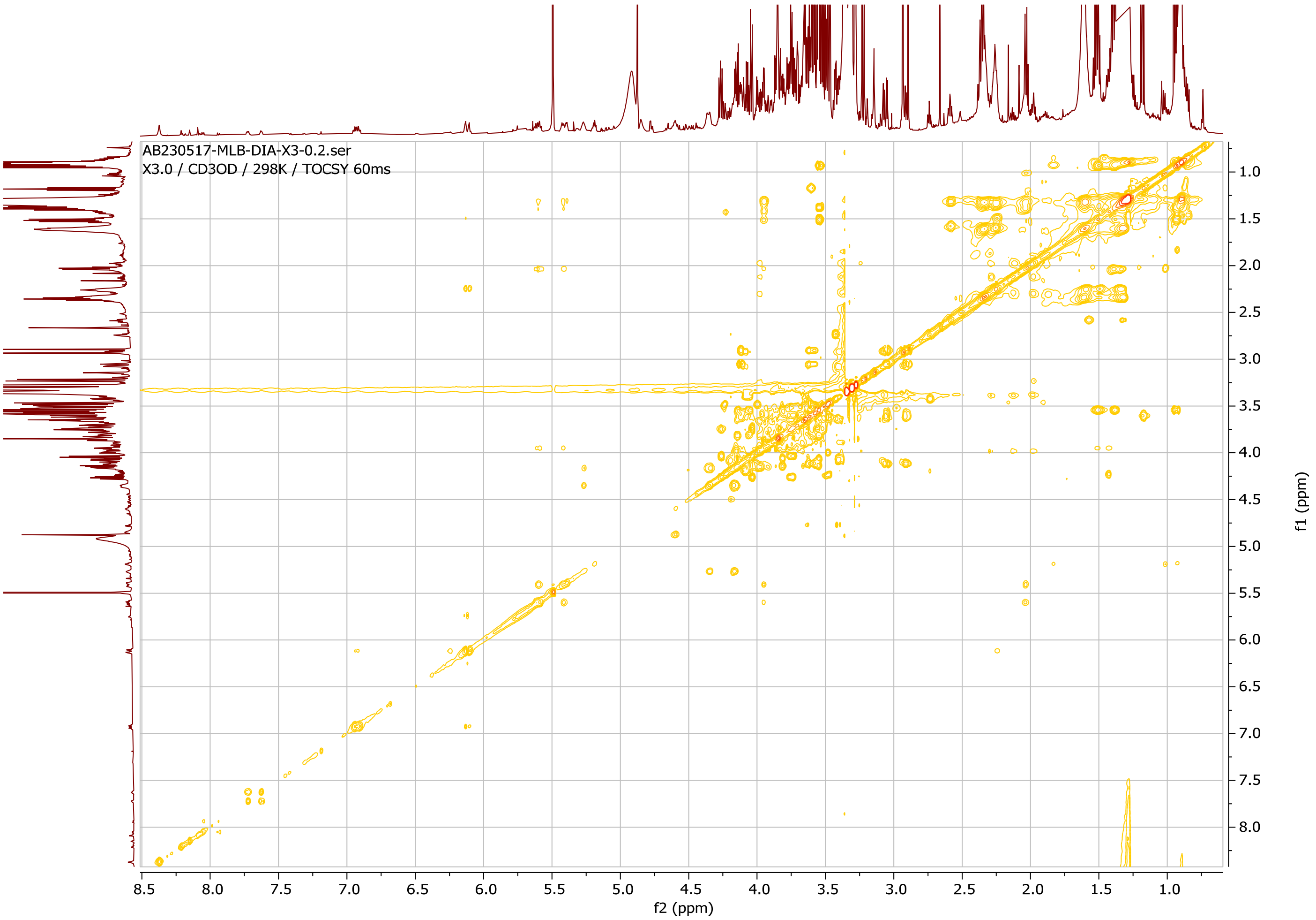

Figure S8: TOCSY-NMR spectrum of the X3.0 planktonic culture (600 MHz, CD_3OD).

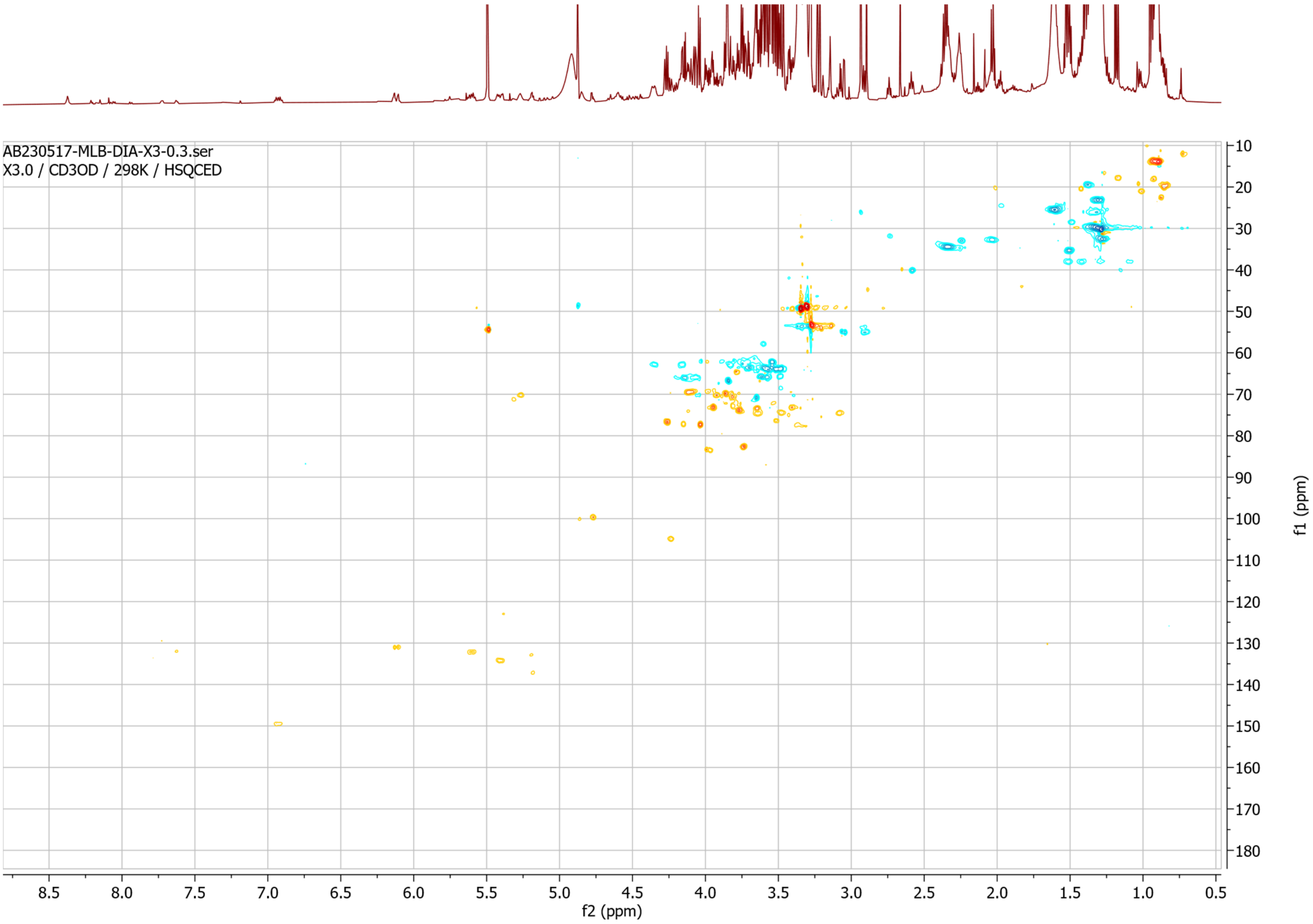

Figure S9: HSQC-ED-NMR spectrum of the X3.0 planktonic culture (600 MHz, CD_3OD).

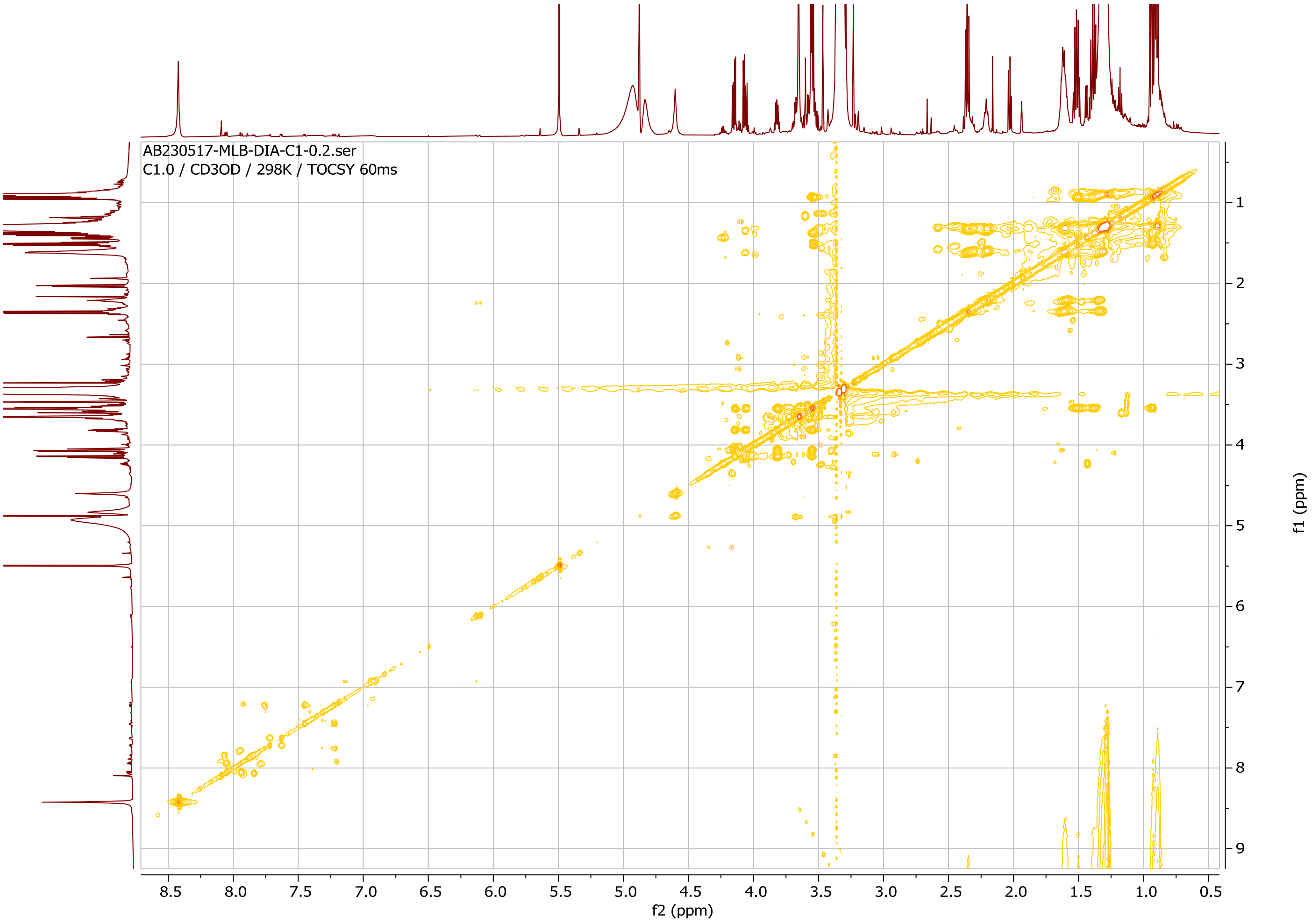

Figure S10: TOCSY-NMR spectrum of the C1.0 CPDMS adherent culture (600 MHz, CD_3OD).

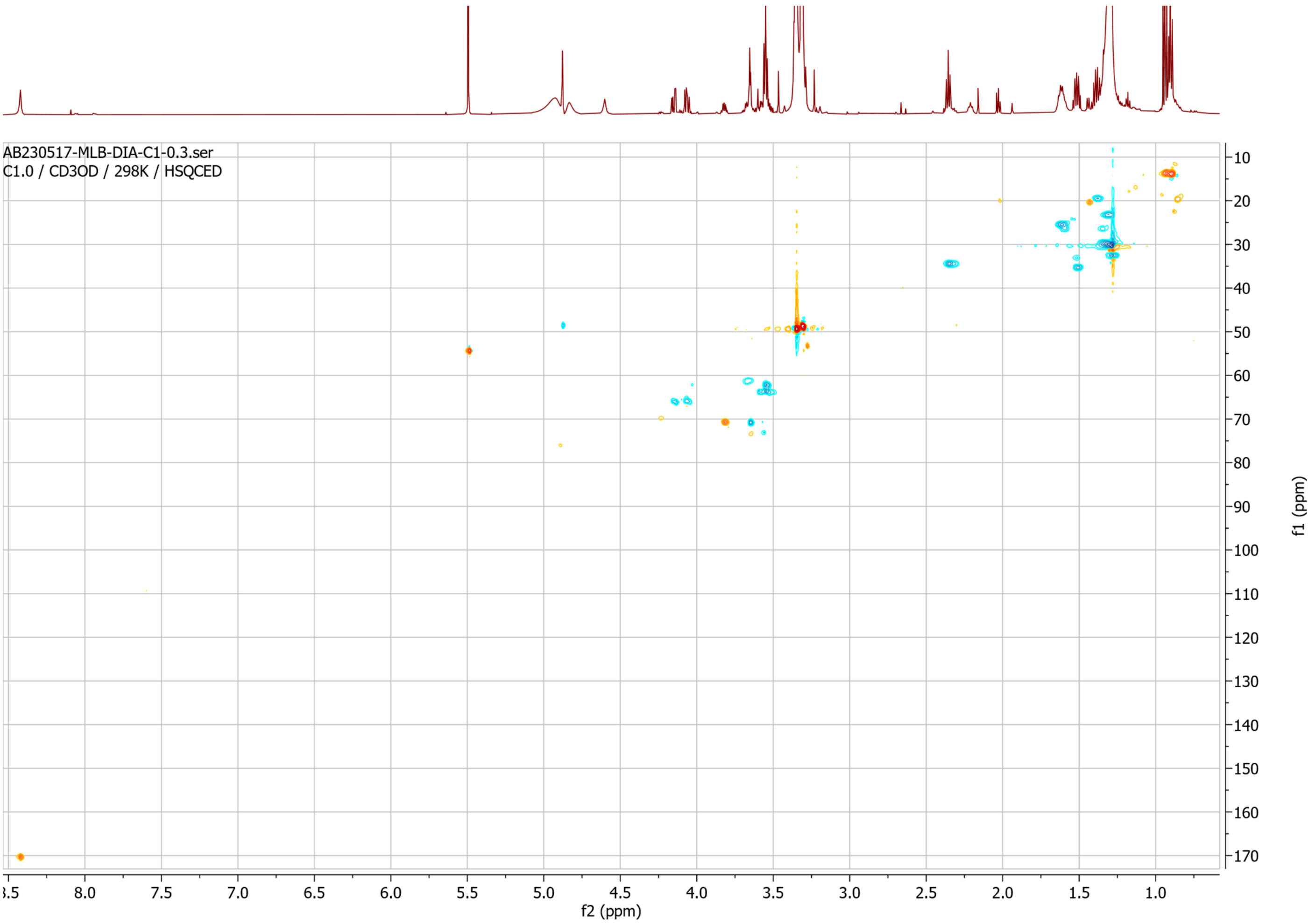

Figure S11: HSQC-ED-NMR spectrum of the C1.0 CPDMS adherent culture (600 MHz, CD_3OD).

Table S1. Discriminants compounds from the LC-HRMS², GC/MS, and NMR analyses on W^{MBPLS} T$_1$

LC/MS discriminant variables	Molecular formula	Molecular name	Pathway	T₁: increased in
LC16	$C_6H_6O_2$	Catechol	Phenylpropanoids and polyketides	CP
LC132	$C_{11}H_{16}O_2$	Dihydroactinidiolide	Terpenoids	CP
LC303	$C_7H_{12}O_6$	Quinic acid	Shikimates and phenylpropanoids	CP
LC650	$C_{13}H_{24}O_{12}$	N.A.	Organic oxygen	CP
LC695	$C_{27}H_{44}O_4$	N.A.	Lipids	CP
LC19	$C_5H_8O_4$	N.A.	Organic acids	CG/CPT/CPDMS
LC41	$C_5H_{10}O_5$	Ribose	Carbohydrates	CG/CPT/CPDMS
LC836	$C_{29}H_{53}NO_9$	DGTA/S 5:1;O2/14:0	Fatty acis	CG/CPT/CPDMS
LC855	$C_{30}H_{55}NO_9$	DGTA/S 6:1;O2/14:0	Lipids	CG/CPT/CPDMS
LC872	$C_{31}H_{57}NO_9$	DGTA/S 7:1;O2/14:0	Lipids	CG/CPT/CPDMS
LC873	$C_{31}H_{57}NO_9$	DGTA/S 5:1;O2/16:0	Lipids	CG/CPT/CPDMS
LC891	$C_{32}H_{59}NO_9$	DGTA/S 8:1;O2/14:0	Lipids	CG/CPT/CPDMS
LC912	$C_{33}H_{61}NO_9$	DGTA/S 9:1;O2/14:0	Lipids	CG/CPT/CPDMS
LC944	$C_{35}H_{65}NO_9$	DGTA/S 9:1;O2/16:0	Lipids	CG/CPT/CPDMS
LC1011	$C_{42}H_{77}NO_8$	DGTA/S 18:2;O1/14:0	Lipids	CG/CPT/CPDMS
LC1024	$C_{44}H_{81}NO_8$	DGTA/S 18:2;O1/16:0	Lipids	CG/CPT/CPDMS

GC/MS discriminant variables	Molecular formula	Molecular name	Pathway	T1: increased in
C10	$C_{16}H_{28}O_2$	(7Z,10Z)-Hexadecadienoic acid (C16:2n-6)	Fatty acids	CP
C18	$C_{20}H_{40}O$	Phytol	Terpenoids	CP
C24	$C_{18}H_{36}O_5$	9,10,12-Trihydroxyoctadecanoic acid (C18:0;O3)	Fatty acids	CP
M16	N.A.	Unknown sugar	Carbohydrates	CP
M42	$C_{12}H_{22}O_{11}$	D-Cellobiose	Carbohydrates	CP
C4	$C_{14}H_{28}O_2$	Tetradecanoic acid (C14:0)	Fatty acids	CG/CPT/CPDMS
M8	$C_5H_{10}O_5$	D-Ribofuranose (isomer 2)	Carbohydrates	CG/CPT/CPDMS
M10	$C_5H_{10}O_5$	D-Ribose	Carbohydrates	CG/CPT/CPDMS
M29	$C_6H_{12}O_6$	*Myo*-Inositol	Carbohydrates	CG/CPT/CPDMS
M33	$C_{18}H_{34}O_2$	(9Z)-Octadecenoic acid (C18:1n-9)	Fatty acids	CG/CPT/CPDMS

NMR discriminant variables		Attribution	Pathway	T1: Increased in
2.90, dd<br>3.04, dd<br>3.62, dd		2,3-dihydroxypropane-1-sulfonate (DHPS)	Organic acids	CP
2.95, s.<br>3.40, t		Dimethylsulfoniopropionate (DMSP)	Organic acids	CP
3.21, s		Choline	Lipids	CP
3.44,		NA		CP
3.75, d<br>3.85, bs<br>3.94, m		Glucose/Galactose	Carbohydrates	CP
0.90, t		Fatty acids CH_3	Lipids	CG/CPT/CPDMS
1.12, t		N.A.		CG/CPT/CPDMS
1.30, bs		Fatty acids CH_2	Lipids	CG/CPT/CPDMS
1.60, t		Fatty acids CH_2 β-ester	Lipids	CG/CPT/CPDMS
2.35, t		Fatty acids CH_2 α-ester	Lipids	CG/CPT/CPDMS
3.54, tt<br>3.82, m<br>4.01, dd<br>4.10, dd		Glycerophospholipids/Glycerolipids	Lipids	CG/CPT/CPDMS
3.65, bs		Glycerol	Lipids	CG/CPT/CPDMS
8.40, s		Formate	Organic acids	CG/CPT/CPDMS

Table S2. Discriminants compounds from the LC-HRMS², GC/MS, and NMR analyses on W^{MBPLS} T_2

LC/MS discriminant variables	Molecular formula	Molecular name	Pathway	T1: increased in
LC718	$C_{18}H_{32}NO_{12}$	DGTA/S 5:1;O2/3:1;O3	Lipids	CG
LC729	$C_{20}H_{34}NO_{11}$	DGTA/S 5:1;O2/5:1;O2	Lipids	CG
LC746	$C_{21}H_{35}NO_{11}$	DGTA/S 5:1;O2/6:1,O2	Lipids	CG
LC762	$C_{22}H_{37}NO_{11}$	DGTA/S 5:1;O2/7:1;O2	Lipids	CG
LC768	$C_{21}H_{37}NO_{12}$	DGTA/S 5:1;O2/6:0,O3	Lipids	CG
LC790	$C_{22}H_{39}NO_{12}$	DGTA/S 5:1;O2/7:0;O3	Lipids	CG
LC812	$C_{25}H_{43}NO_{11}$	DGTA/S 6:1;O2/9:1;O2	Lipids	CG
LC885	$C_{31}H_{53}NO_{10}$	DGTA/S 5:1;O2/16:2;O	Lipids	CG

GC/MS discriminant	Molecular formula	Molecular name	Pathway	T2: increased in

variables				
C19	$C_{18}H_{28}O_2$	(6Z,9Z,12Z,15Z)-Octadecatetraenoic acid (C18:4n-3)	Fatty acids	CG
C26	$C_{22}H_{32}O_2$	4,7,10,13,16,19-Docosahexaenoic acid (C22:6n-3)	Fatty acids	CG
C9	$C_{21}H_{44}$	2,6,10,15-Tetramethylheptadecane	Alkane	CP/CPT/ CPDMS
M3	$C_3H_8O_3$	Glycerol	Lipids	CP/CPT/ CPDMS
M28	$C_8H_{15}NO_6$	*N*-Acetyl-D-Glucosamine (isomer 1)	Carbohydrates	CP/CPT/ CPDMS
**NMR** discriminant variables		**Attribution**	**Pathway**	**T_2: Increased in**
1.01, d		Isoleucine	Amino-acids	CG
3.21, s		Choline	Lipids	CG
4.12, dd. 4.19, dd		Sulphoquinovosyldiacylglycerols (SQDGs)	Lipids	CG
6.10, dd		N.A.		CG
6.92, dd		N.A.		CG
0.74, t		Sterols	Lipids	CG
1.03, d		Valine	Amino-acids	CG
1.60, t		Fatty acids CH_2 β ester	Lipids	CG
2.32, m		Proline	Amino-acids	CG
2.60, t		Fatty acids CH_2 α ester	Lipids	CG
2.82, s		N.A.		CG
1.45, d		Lactate	Glycolysis	CPT/CPDMS/CP
3.65, bs		Glycerol	Lipids	CPT/CPDMS/CP

Table S3. Discriminants compounds from the LC-HRMS², GC/MS, and NMR analyses on W^{MBPLS} T_3

**LC/MS** discriminant variables	**Molecular formula**	**Molecular name**	**Pathway**	**T_3: increased in**
LC52	N.A.	$C_8H_{16}N_2$	N.A.	CPT
LC73	N.A.	$C_8H_8N_2O$	Alkaloids	CPT
LC241	N.A.	$C_{13}H_{24}N_2O$	Alkaloids	CPT
LC315	N.A.	$C_{14}H_{24}N_2O_2$	Alkaloids	CPT
LC1039	Triglyceride (AG:14:0/16:0/16:2)	$C_{49}H_{88}O_6$	Lipids	CPDMS
**GC/MS** discriminant variables	**Molecular formula**	**Molecular name**	**Pathway**	**T_3: increased in**
C8	2-Methyloctadecane	$C_{19}H_{40}$	Alkane	CPT
C12	(9*Z*)-Hexadecenoic acid (C16:1n-7)	$C_{16}H_{30}O_2$	Fatty acids	CPT
M26	D-Glucuronic acid	$C_6H_{10}O_7$	Carbohydrates	CPT
M36	D-Glucose	$C_6H_{12}O_6$	Carbohydrates	CPT
C16	Isophytol, acetate	$C_{22}H_{42}O_2$	Terpenoids	CPT
**NMR** discriminant variables		**Attribution**	**Pathway**	**T_1: Increased in**
0.90, t		Fatty acids CH_3	Lipids	CPT
1.30, bs		Fatty acids CH_2	Lipids	CPT
1.60, t		Fatty acids CH_2 β ester	Lipids	CPT
2.35, t		Fatty acids CH_2 α ester	Lipids	CPT
4.10, dd		Glycerophospholipids/Glycerolipids	Lipids	CPT
